# Pooling breast cancer datasets has a synergetic effect on classification performance and improves signature stability

**DOI:** 10.1186/1471-2164-9-375

**Published:** 2008-08-06

**Authors:** Martin H van Vliet, Fabien Reyal, Hugo M Horlings, Marc J van de Vijver, Marcel JT Reinders, Lodewyk FA Wessels

**Affiliations:** 1 Information and Communication Theory Group, Faculty of Electrical Engineering, Mathematics and Computer Science, Delft University of Technology, Mekelweg 4, 2628 CD Delft, The Netherlands; 2 Bioinformatics and Statistics group, Department of Molecular Biology, Netherlands Cancer Institute, Plesmanlaan 121, 1066 CX Amsterdam, The Netherlands; 3 Department of Pathology, Netherlands Cancer Institute, Plesmanlaan 121, 1066 CX Amsterdam, The Netherlands; 4 Department of Pathology, Academic Medical Center, Meibergdreef 9, 1100 DD, Amsterdam, The Netherlands; 5 Department of Surgery, Institut Curie, 6 rue d'Ulm, 75005 Paris, France

## Abstract

**Background:**

Michiels *et al. *(Lancet 2005; 365: 488–92) employed a resampling strategy to show that the genes identified as predictors of prognosis from resamplings of a single gene expression dataset are highly variable. The genes most frequently identified in the separate resamplings were put forward as a 'gold standard'. On a higher level, breast cancer datasets collected by different institutions can be considered as resamplings from the underlying breast cancer population. The limited overlap between published prognostic signatures confirms the trend of signature instability identified by the resampling strategy. Six breast cancer datasets, totaling 947 samples, all measured on the Affymetrix platform, are currently available. This provides a unique opportunity to employ a substantial dataset to investigate the effects of pooling datasets on classifier accuracy, signature stability and enrichment of functional categories.

**Results:**

We show that the resampling strategy produces a suboptimal ranking of genes, which can not be considered to be a 'gold standard'. When pooling breast cancer datasets, we observed a synergetic effect on the classification performance in 73% of the cases. We also observe a significant positive correlation between the number of datasets that is pooled, the validation performance, the number of genes selected, and the enrichment of specific functional categories. In addition, we have evaluated the support for five explanations that have been postulated for the limited overlap of signatures.

**Conclusion:**

The limited overlap of current signature genes can be attributed to small sample size. Pooling datasets results in more accurate classification and a convergence of signature genes. We therefore advocate the analysis of new data within the context of a compendium, rather than analysis in isolation.

## Background

Various gene expression signatures have been extracted from breast cancer microarray data [1,2] to predict outcome. Subsequently, these signatures have been validated in larger cohorts [3,4], the datasets were re-analyzed to assess their robustness [5], or other data mining techniques were tested [6,7]. Depending on the technique used, different signatures are extracted from the same dataset. Often, these signatures have varying degrees of overlap in genes that compose the signature [8]. This has led to the insight that there is no unique signature [9].

From a clinical point of view, accuracy of a signature is very important [10]. To extract such an accurate gene expression signature, which is predictive of outcome, a variety of methodologies have been proposed [11,12]. Typically, the group of patients is dichotomized based on a five year survival threshold, resulting in a poor and a good outcome group. This assignment then serves as a class label, and the aim of this work is to predict this label. Most protocols apply a cross validation scheme, with which the generalization error and optimal number of genes are estimated. A final classifier is obtained by selecting the optimal number of genes and training the final classifier on all samples.

Michiels *et al. *[5] introduced the concept of repeated random resampling to estimate the robustness (stability) of proposed signatures. The strategy relies on repeatedly analyzing a subset of patients in a dataset (a resampling), and then combining the results of the separate resamplings into a final list of genes. This strategy has been employed to investigate the stability of proposed signatures, and produces, by repeated resampling, a gene list which is employed as a type of 'gold standard'. This 'gold standard' has been shown to be different from signatures previously reported on the same data [5,13,14].

However, the repeated random resampling approach has several drawbacks. First, resampling selects a subset of the available data, thereby reducing the number of samples and intensifying the small sample size problem. Second, the actual ranks within each resampling are not used (only presence in the top *N *genes is employed as a quality measure), which implies that the available information is not fully exploited. Lastly, the true rank of genes is unknown, making it hard to show that this strategy indeed provides a better ranking.

We set up an experiment using artificial datasets to test the resampling strategy in a controlled environment. This allowed us to compare the resampling based ranking to the true ranking. In this comparison, we included two variants of the resampling strategy, and a non-resampling based strategy. On a higher level, datasets from different institutions consisting of samples from the same disease type, can be viewed as resamplings from the same underlying population. Concurrent analysis of these datasets aimed at, for example, producing a classifier, can be performed using a range of integration strategies. From a statistical point of view, a larger dataset implies more statistical power, advocating an early integration approach where (normalized) datasets are pooled prior to the analysis [6]. A classifier derived from such a pooled dataset, should, in theory, be superior to those derived from a single dataset. Of course, in order to minimize inter-dataset variability, one of a variety of normalization techniques should be employed (e.g. Z-score normalization per feature per dataset) prior to pooling the datasets. Alternatively, a rank-based classification methodology has been put forward [15,16], which is independent of dataset normalization. Moreover, heterogeneity in the different datasets might be a hurdle [17], which might not be overcome with simple normalization approaches. In such cases, performing a meta-analysis may be advantageous, which implies combining statistics from each dataset. Lastly, 'late integration' (also termed 'combining decisions') involves training a classifier on the separate datasets and then merging the separate decisions to reach a final result. In fact, this approach possesses the least statistical power, since each of the individual classifiers have no benefit from the other datasets.

A setting using artificial datasets, allows for a controlled analysis of the effects observed when pooling these artificial datasets. If synergy exists between the artificial datasets, we hypothesize that the classifier performance should improve when more artificial datasets are pooled. Therefore, we extended our analysis on artificial data to multiple artificial datasets. First of all, we start pooling pairs of artificial datasets, and thereafter increase the number of pooled datasets pooled until all six artificial datasets are pooled. We explored the effect of pooling artificial datasets on the classification performance (double loop cross validation), independent validation, and signature size.

Currently, multiple breast cancer datasets are publicly available [1,2,18,19]. Teschendorff *et al. *[20] created a consensus ER positive classifier from three breast cancer datasets employing a meta-analysis strategy.

These datasets were measured on different platforms, thereby introducing difficulties with respect to probe matching, and the fact that different reference pools may have been used. If direct pooling of the datasets would be allowed, such a meta-analysis would not yield the optimal result. Moreover, true synergy of the meta-analysis on multiple datasets wasn't shown. Similarly, Xu *et al. *[21] have created a consensus rank-based classifier from three different datasets, which was validated on a fourth independent dataset. To validate the type of effects observed when pooling artificial datasets, we applied the same analysis to a compendium of six breast cancer datasets (Desmedt *et al. *[4], Minn *et al. *[22], Miller *et al. *[18], Pawitan *et al. *[23], Loi *et al. *[24], Chin *et al. *[25]). This compendium of breast cancer datasets is particularly suited for such an analysis, since they were all measured on the same platform (Affymetrix HG U133A). In this setup, we inspected the effect of pooling breast cancer datasets on the classification performance (double loop cross validation), independent validation (a seventh dataset), signature size, and functional enrichment.

Lastly, the analysis on such a compendium of six breast cancer datasets, might provide answers to an important unsolved question: why is there only limited overlap between existing signatures? [8] As potential explanations, several explanations have been postulated, none of which have up to now been rigorously tested. We employ the results obtained when progressively pooling more breast cancer datasets, to provide insight into the correctness of the five explanations, and identify sample size as the most likely cause of limited signature overlap.

## Results and discussion

### Resampling on a single breast cancer dataset

**Figure 1:**
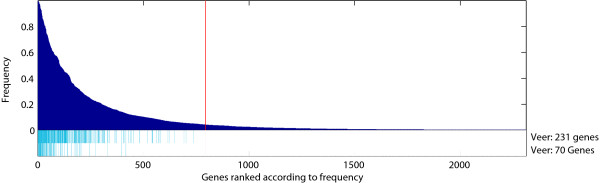
**Result of the repeated random resampling procedure on the Veer *et al.***[1]** data.** The histogram shows the frequencies of genes being among the top 200 genes over 500 resamplings. Below the histogram, two lanes containing light-blue bars indicate the genes that are part of the published signatures. The red line indicates the frequency threshold corresponding to the expected value of the frequency under the null hypothesis (no information) given the number of genes *N *that is selected in each of the *R *resamplings.

We applied the repeated random resampling strategy (see Methods section) on the Veer *et al. *[1] dataset, see Figure 1. When comparing the frequency-based ranking to the set of genes in the published 70-gene and 231-gene signatures, there appear to be additional genes with high frequencies of occurrence that were not part of the published signatures. Both Michiels *et al. *[5], and Roepman *et al. *[13] point out that the genes with high frequencies of occurrence, which are not part of the original signature, are of high interest. They hypothesize that these genes were probably not picked up in the signatures due to sampling specific biases in the dataset. This suggests a beneficial effect due to the resampling which does pick them up. However, since the true ranking of genes underlying breast cancer outcome is unknown, a plausible alternative hypothesis could be that those genes are false positives.

Roepman *et al. *[13] considers all genes with a non-zero frequency of occurrence to represent a robust signature. This seems rather optimistic, since genes will be expected to have a positive frequency by chance. Therefore, we propose to set a threshold to prune the set of genes to only those with a frequency higher than expected by chance (see Methods section). This threshold is indicated by the vertical red line in Figure 1. The set of genes retained after this pruning step still contains all genes from the original signatures, i.e. all light-blue bars (the original signatures) reside on the left of the threshold. Of course, the ranking itself remains unchanged.

In the current experimental setting, it is impossible to conclude which hypothesis correctly describes the appearance of genes with a high (above the threshold) frequency of occurrence, that were not in the original signature.

### Resampling on a single artificial dataset

We set up an experiment on artificial data to be able to compare the ranking after resampling to a known ground truth (see Methods section). The artificial dataset mimics a microarray dataset, and consists of 5000 genes, of which either 500, 2500 or 5000 genes were informative. The experiments were done using artificial datasets consisting of 100 to 1000 samples, with equal priors for the two classes. The data was sampled from a class-conditional gaussian distribution, without any covariance structure. Since genes are assumed to be independent, these datasets are more ideal than real microarray datasets. Nevertheless, this controlled, artificial setting can be a useful tool to gain insight into the relationship between sample size and gene selection.

**Figure 2:**
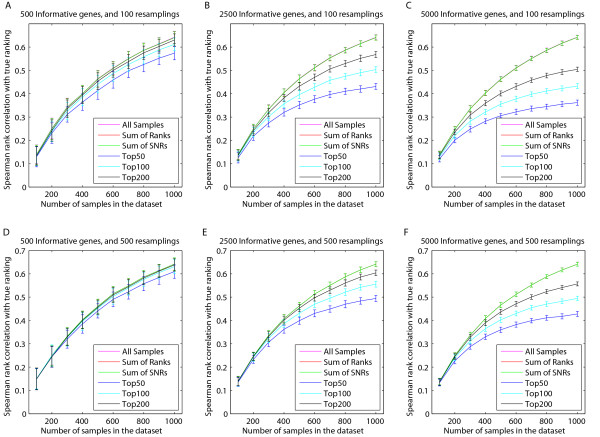
**Spearman rank correlation of the ranking obtained after resampling an artificial dataset and the true ranking.** The number of informative genes was varied from 500 (A and D), 2500 (B and E), to 5000 (C and F), and 100 (A, B and C) or 500 (D, E and F) resamplings were considered. The errorbars indicate the mean and standard deviation over 100 repeats of the entire experiment. The results from the 'All Samples', 'Sum of Ranks', and 'Sum of SNRs' methods are equivalent, and are therefore plotted on top of each other (top line in all plots).

On these artificial datasets we compare the ranking resulting from the resampling strategy with the ranking based on all samples. Additionally, we also consider two variants of the resampling strategy. Instead of only storing the top *N *genes in each of the resamplings, we sum the statistics (sum of ranks or sum of signal-to-noise ratios (SNRs)) of the genes across all the resamplings, and base the final ranking on this cumulative statistic. Summing the statistics amounts to a 'meta-analysis', and the Top *N *method is a 'combining decisions' approach. We then compare the ranking obtained by each method to the ground truth (Spearman rank correlation). Since a particular initialization of the artificial data might influence the results, we repeated the entire experiment 100 times, and present statistics across all repeats (average and standard deviation). Figure 2 shows the Spearman rank correlation between the known ranks of the artificial data and those obtained by the Top *N *resampling approaches, the two resampling variants, and the ranking over all samples. For *N *we considered values of 50, 100 and 200, similar to Michiels *et al. *[5], and Roepman *et al. *[13].

Figure 2 indicates that the ranking based on all samples outperforms the ranking obtained from resampling and then determining the frequency of being in the top *N*. Furthermore, it is evident that combining the statistics (sum of ranks or sum of SNRs) of each resampling performs similar to the ranking over all samples. In fact, it is quite straight-forward to show analytically that, for a given gene, the summation of SNRs across resamplings is equal to the SNR over all samples (see Additional file 1). This is true when enough resamplings are performed, and they are uniformly sampled from the complete dataset.

For smaller artificial datasets the obtained Spearman rank correlations are lower compared to larger artificial datasets. The reduced sample size is the most plausible explanation for this effect. For larger artificial datasets, the gap in performance between the top *N *resampling approaches and the sum of ranks and sum of SNRs widens, indicating that the latter two methods profit more from an increase in artificial dataset size.

Taken together, these results clearly show that the random resampling strategy produces a suboptimal ranking of the prognostic genes, and that it is preferable to employ a ranking based on all samples in the dataset. In addition, these differences become more pronounced as the sample size increases. Given that these effects are already evident on an artificial dataset, we hypothesize that the sample size effects will be even stronger on data with a complex covariance structure. We therefore conclude that it is unwise to use the repeated random resampling approach on real data for ranking features.

### Pooling artificial datasets

The experiment on a single artificial dataset is easily extendible to a scenario where multiple artificial datasets are available. Such a setup would emulate the availability of several microarray datasets from the same biological context. We hypothesize that a significant association exists between the classification error and the number of datasets pooled.

**Figure 3:**
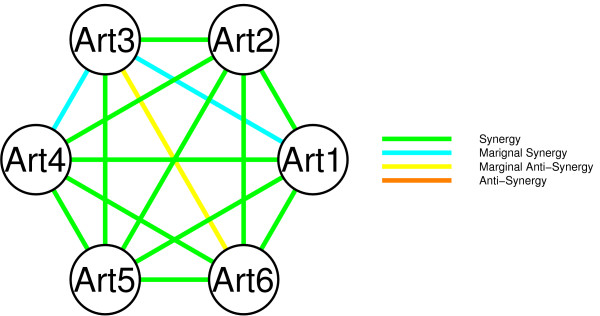
**Network indicating the synergy between six artificial datasets (Art1 to Art6).** Each of these six datasets were generated from the same model, without introducing any noise or heterogeneity. Each node represents a dataset, and each edge the effect on the DLCV error when pooling them. Four different effects were considered, synergy (bright green) when the pooled error is lower than each of the separate errors. Marginal synergy (light blue) when the pooled error is lower than the weighted mean of the separate errors, conversely marginal anti-synergy (yellow) when it is higher. Lastly, true anti-synergy (orange) indicates a higher DLCV error for the pooled dataset.

We set up an experiment using six artificial datasets, each with 200 samples and 5000 genes, of which 500 are informative. Each of these six artificial datasets were generated using the same model, and no additional noise or heterogeneity was introduced. This represents the ideal case, where each of the six datasets are truly relevant for the same underlying problem. We applied the double loop cross validation (DLCV, nearest mean classifier) protocol on each artificial dataset separately (see Methods section), and each possible pooled subset of artificial datasets. First of all, we inspected the behavior of pooling pairs of artificial datasets relative to the performance on the individual artificial datasets. A DLCV error which is lower for the pooled pair compared to the individual errors implies a synergetic effect on the classification performance. When the pooled DLCV error is between the two individual errors, and lower/higher than the weighted mean (number of samples chosen as weights) we label the effect as marginal synergetic/marginal anti-synergetic. Conversely, if the DLCV error is strictly higher than the individual errors it is labeled as anti-synergetic. Figure 3 shows a network indicating the type of synergy that was observed for the six artificial datasets, labeled Art1 to Art6.

Figure 3 shows that there is a synergetic effect on the DLCV error for 14 of the 15 pooled pairs of artificial datasets. A marginal anti-synergetic effect is only observed in one of the 15 cases. This suggests a large gain in statistical power when pooling the artificial datasets.

**Figure 4:**
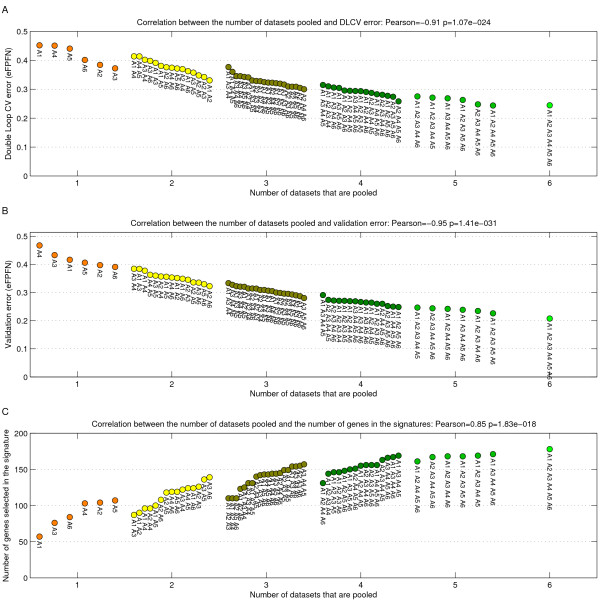
**Scatterplot indicating the classification error relative to the number of datasets that is pooled.** A) DLCV error. B) Error on a large independent validation set of 2000 samples. C) Number of genes selected by the DLCV protocol. The color corresponds to the number of datasets that was used. Poolings with the same number of datasets are sorted based on error/number of genes. Labels indicate which combination of datasets was used.

Next, we evaluated the DLCV error for every potential pooling of one to six artificial datasets. In addition, we evaluated the performance of each of the classifiers derived from the pooled data on an independent large validation set of 2000 artificial samples, see Figure 4A and 4B. Figure 4A and 4B show that a high degree of synergy is obtained by pooling artificial datasets. Both the DLCV error as well as the error on the validation set show a highly significant correlation with the number of artificial datasets that is pooled, Pearson correlation of -0.91 (*p *= 1.1*e *- 24) and -0.95 (*p *= 1.4*e *- 31), respectively.

To test whether the observed trends are classifier specific, we repeated the experiment using non-linear classifiers (as opposed to the linear nearest mean classifier used above). Thus, we repeated the experiment using a K-Nearest Neighbor classifier (K-NN) as well as a Support Vector Machine with a Radial Basis Function as kernel (SVM with RBF kernel). Using these classifiers, we observed the same trends for the DLCV and Validation error. That is, in all cases the performance gets better after pooling the artificial datasets (trends significant, *p *< 0.05). However, the errors obtained are higher than those from the NMC. The plots are shown in Additional files 2 and 3.

In addition to the classification performance, we looked at the optimal number of genes that is selected by the classifiers, see Figure 4C. It turns out that there is a highly significant positive trend (Pearson correlation 0.85, *p *= 1.8*e *- 18) towards selecting more genes when artificial datasets are pooled.

In summary, pooling artificial datasets shows that larger training sets result in larger gene sets being selected and, most importantly, in better classification performance. Since this effect is already clearly present in the simple model, we believe that this effect will be even stronger in more complex models which include, for example, a covariance structure between genes. This stems from the fact that these more complex models will require more data (relative to the simple models) to accurately estimate the required complex classifiers.

### Pooling breast cancer datasets

**Figure 5:**
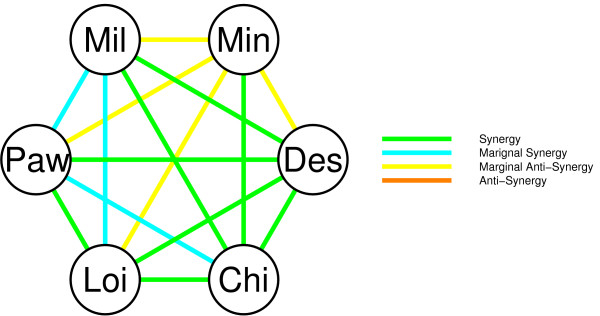
**Network indicating the synergy between six real datasets.** Each node represents a dataset, and each edge the effect on the DLCV error when pooling them. Four different effects were considered, synergy (bright green) when the pooled error is lower than each of the separate errors. Marginal synergy (light blue) when the pooled error is lower than the weighted mean of the separate errors, conversely marginal anti-synergy (yellow) when it is higher. Lastly, true anti-synergy (orange) indicates a higher DLCV error for the pooled dataset.

We used a compendium of six Affymetrix breast cancer datasets to evaluate the effect of pooling datasets on the performance of classifiers (see Table 1 and methods section). Our aim is to classify patients into the poor/good outcome groups as well as possible. To this end, we followed the same strategy as on the artificial datasets. First, we inspected the synergy effect on the DLCV error achieved on pairs of datasets relative to the DLCV errors on the individual datasets (DLCV, nearest mean classifier). Figure 5 shows a network indicating the synergy among the six real datasets.

**Table 1:** Indication of the origin of the seven datasets that were used.

				All ER		ER pos		ER neg	
Publication:	Label	Survival	Total	Poor	Good	Poor	Good	Poor	Good
Desmedt *et al. *[4]	Des (D)	DMFS	120	29	91	16	65	13	26
Minn *et al. *[22]	Min (Mn)	DMFS	62	21	41	9	30	12	11
Miller *et al. *[18]	Mil (Ml)	SOS	193	37	156	26	117	11	39
Pawitan *et al. *[23]	Paw (P)	SOS	142	22	120	14	99	8	21
Loi *et al. *[24]	Loi (L)	DMFS	120	28	92	21	71	7	21
Chin *et al. *[25]	Chi (C)	DMFS	86	23	63	14	50	9	13
Vijver *et al. *[3]	Vij (V)	DMFS	248	70	178	44	149	26	29

The Survival column indicates the type of survival data that was used; DMFS Distant Metastasis Free Survival; SOS breast cancer Specific Overall Survival. Lastly, for each dataset the total number of samples is indicated along with the number of poor/good samples per ER subgroup.

Figure 5 shows that for 11 of the 15 pairwise pooled datasets a synergetic effect was observed (green/blue link). For 4 of the 15 pairwise pooled datasets there was a marginal anti-synergetic effect (yellow link), and for none of the pairs the effect was anti-synergetic (orange link).

**Figure 6:**
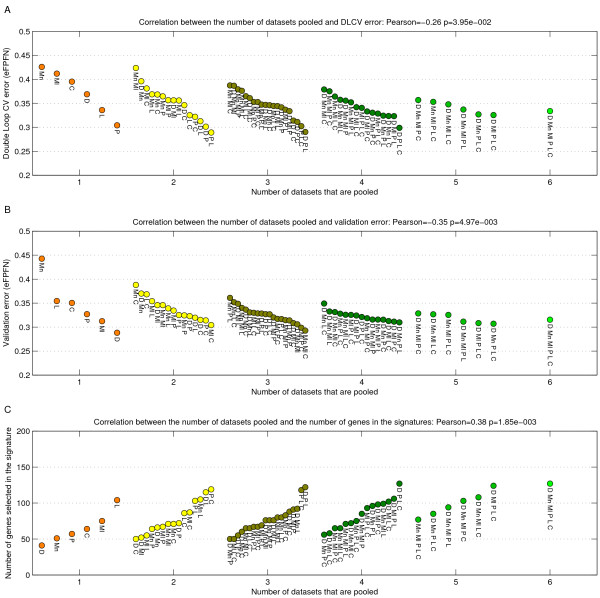
**Scatterplot indicating the classification error relative to the number of datasets that is pooled.** A) DLCV error. B) Error on the Vijver *et al. *[3] dataset. C) Number of genes selected by the DLCV protocol. The color corresponds to the number of datasets that was used. Poolings with the same number of datasets are sorted based on error/number of genes. Labels indicate which combination of datasets was used.

As a next step we applied the DLCV framework on each potential pooling of the datasets, and assessed the DLCV error. In addition, we evaluated the performance of each classifier on a seventh independent dataset (Vijver *et al. *[3]). Figure 6A and 6B show the DLCV error and validation error as a function of the number of datasets pooled.

There is a clear trend indicating that the DLCV error decreases when more datasets are pooled (see Figure 6A). The observed trend has a Pearson correlation of -0.26, which is significant: *p *= 4.0*e *- 2. Of course, since the different datasets have different numbers of samples, an alternative would be to evaluate the correlation of the error and the actual number of samples in the pooled datasets. Doing this unveils a similar significant correlation (Additional file 4).

The results on the seventh independent validation set (Vijver *et al. *[3] data) confirm the synergetic effect obtained by pooling datasets (see Figure 6B). The trend is even more pronounced at a Pearson correlation of -0.35, with *p *= 5.0*e *- 3. In addition, in a multivariate Cox proportional hazards model the signature is an independent predictor of outcome in the presence of the standard clinico-pathological variables (data not shown). Similarly, plotting the results in a Kaplan-Meier plot indicates a highly significant separation (*p *= 9.5*e *- 7, logrank test, data not shown).

The nearest mean classifier that was used in the above experiment is a relatively simple linear classifier, which is known to perform well on this type of data [26]. To test whether the observed trend is classifier specific, we repeated the experiment using non-linear classifiers. Thus, we repeated the experiment using a K-Nearest Neighbor classifier (K-NN) as well as a Support Vector Machine with a Radial Basis Function as kernel (SVM with RBF kernel). The results that were obtained for the DLCV error and the validation error on the Vijver dataset are shown in Additional files 5 and 6, for the K-NN and SVM classifiers, respectively.

Overall, the average eFPFN across all 63 different pooling combinations when using the K-NN classifier (DLCV = 0.356) is slightly worse compared to the nearest mean classifier (DLCV = 0.348). In addition, the error obtained using the SVM (DLCV = 0.391) is higher than the K-NN. Similarly, the average error on the independent validation set (Vijver) is also higher: (0.329 for the NMC, 0.365 for the K-NN and 0.405 for the SVM). This corresponds to the previous observation that simpler classifier often perform better on this type of data. At the same time, the trend that classifiers that are derived from pooled datasets are more accurate is also observed.

In conclusion, not only simple linear classifiers, but also more complex non-linear classifiers show a clear benefit by training them on pooled datasets. Moreover, these results confirm that the linear nearest mean classifier is the best candidate for this type of classification problem.

Previously reported classifiers have been using varying numbers of genes. Signatures that have been derived using a cross validation setup that is similar to the one used here, use 52 [20] to around 70 genes [2,3]. We have investigated the number of genes selected by the DLCV method, for each of our pooled datasets, see Figure 6C. The classifiers trained on a single dataset use around 50 genes, whereas pooled datasets select progressively more genes. Especially the classifiers from five or six datasets select more than 100 genes. These results indicate a positive trend in the number of datasets pooled and the number of genes selected in the signatures (Pearson correlation of 0.38 with *p *= 1.9*e *- 3).

In summary, the effects observed when pooling artificial datasets are also observed when pooling breast cancer datasets. This implies that datasets should be analyzed jointly rather than in isolation.

### Enrichment of the signatures

Functional enrichment of prognostic signatures has unveiled a multitude of pathways that are associated with tumor progression. We set out to inspect the effect of pooling datasets on the enrichment. We used a collection of 1860 genes sets consisting of at least five annotated genes (see Methods section). For each pooled combination, we used the hypergeometric test to assess the significance of the overlap, followed by Bonferroni correction (see Methods section).

**Figure 7:**
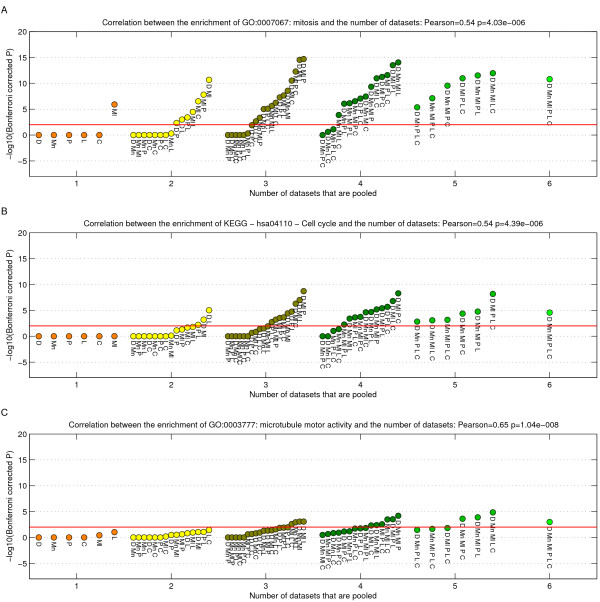
**Enrichment of three gene sets relative to the number of datasets which is pooled.** A) GO:0007067: mitosis B) KEGG – hsa04110 – Cell cycle C) GO:0003777: microtubule motor activity. Scatterplots indicate the minus log10 of the Bonferroni corrected p-values. The red line indicates the level at which 0.01 is reached.

**Figure 8:**
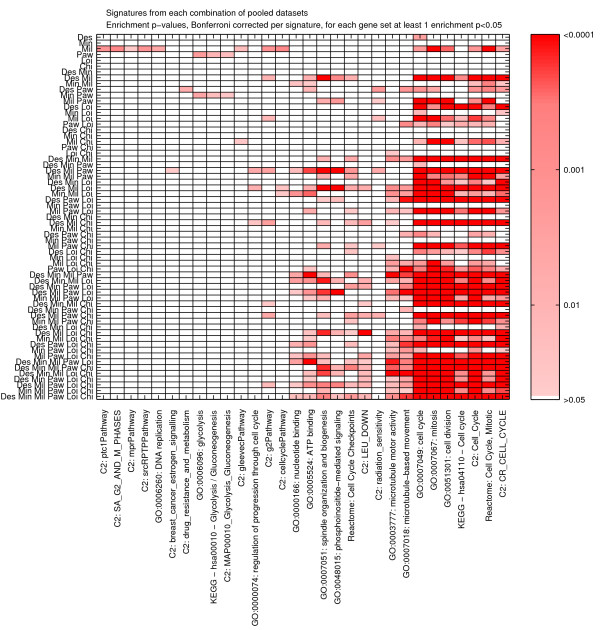
**Heatmap of the Bonferroni corrected p-values of the enrichment between each signature and a collection of gene sets.** Only categories with at least 1 significant association are shown.

The most highly enriched categories were proliferation associated, which are well-known to be associated with tumorigenesis. Figure 7 shows the enrichment for three of the gene sets. Figure 8 depicts a complete heatmap of enrichments.

As seen in Figure 7A, the mitosis GO category (GO:0007067: mitosis) shows a significant enrichment for practically every pooled combination of datasets. This is an example of a very strong signal, which gets even stronger when pooling datasets. Another proliferation related category (Figure 7B), the cell cycle pathway (KEGG – hsa04110 – Cell cycle) becomes significantly enriched when roughly three or more datasets are combined. Thus it's association is weaker than the mitosis gene set, but gets detected when pooling a few datasets.

Lastly, a gene set associated to microtubule activity (GO:0003777: microtubule motor activity) is non-significant for the range of pooling one to three datasets (Figure 7C). When combining four or five it is sometimes picked up, and is definitely picked up when pooling six datasets. This is a clear example of a relatively weak signal, which benefits significantly from pooling datasets.

Conversely, we also observe that many gene sets which are significant in some of the single/pairs of datasets are no longer significant when pooling (Figure 8). For instance, glycolysis related pathways are only picked up in the signature extracted from the Pawitan *et al. *[23] dataset. This is evidence that those categories are specific to this dataset, but represent false positives within the context of the global breast cancer population.

Overall, the enrichment analysis provides further proof that pooling datasets significantly increases the statistical power to detect weak signals and filter out false positive associations.

### Heterogeneity amongst the breast cancer datasets

Pooling datasets with a high degree of heterogeneity might have a detrimental effect on subsequent analysis. A potential way to limit the heterogeneity would be to restrict the analysis to known clinical subgroups. To test this, we split each dataset based on ER status, and repeated the analysis on each of these subgroups. Of course, these splits reduce the number of samples and intensify small sample size related problems. For this reason it becomes impractical to exhaustively explore all possible stratifications of the dataset. We chose the ER status since it is known to have a profound effect on disease progression and gene expression.

On the subgroup of ER positive samples, we observed the same trends as on the complete datasets. We observed a synergetic effect for 10 of the 15 of the pooled pairs of datasets (see Additional file 7). Moreover, the trends observed on all samples are also significant on the ER positive subset (see Additional file 8). Enrichment analysis of these signatures revealed the same set of categories that were found on the complete set of samples (see Additional file 9).

Similarly, we also repeated all analyses on the ER negative subgroup of samples. We observe the same trends as on all samples or the ER positive group (see Additional file 10 and 11). However, the classification accuracy on this subgroup is much lower. A potential explanation for this observation is that this group of samples is inherently more difficult to classify, or that the small sample size related problems play a big role (combining all six datasets results in 191 samples).

Enrichment analysis of the signatures from the ER negative group, leads to a very limited set of enriched gene sets (see Additional file 12). The most highly enriched category is 'GO:0006955: immune response'. This confirms previous reports on the association of this category with outcome in the ER negative group [27,28]. However, in the largest dataset (all ER negative samples), not a single category is enriched. In both of the ER groups, we observed the same synergetic trends when pooling datasets on cross validation, independent validation, and the number of genes selected. Therefore, heterogeneity among the datasets due to ER status is only limited.

### Overlap amongst signatures

It has been previously observed that existing signatures have limited overlap in terms of genes. Several potential explanations have been proposed for these differences:

1. Heterogeneity in expression due to different platform technologies and references;

2. Differences in supervised protocols with which signatures are extracted;

3. Although the genes are not exactly the same, they represent the same set of pathways;

4. Differences in clinical composition between datasets (i.e. sample heterogeneity) and

5. Small sample size problems that cause inaccurate signatures.

Here we will critically evaluate whether we observe support for each of these explanations in the context of the six breast cancer datasets we studied.

#### Explanations 1 and 2

**Figure 9:**
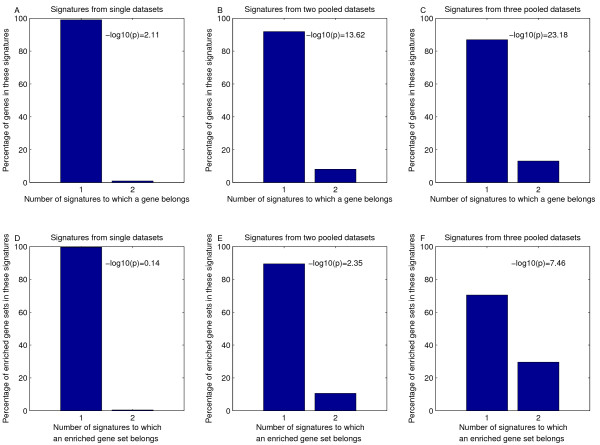
**Histograms indicating the percentage of genes (A-C) and enriched gene sets (D-F) that overlap between two signatures.** A and D) Median histogram and hypergeometric p-value across every pairwise comparison of signatures from single datasets. B and E) Median histogram and hypergeometric p-value across every pairwise comparison of signatures from 2 pooled datasets C and F) Average histogram and hypergeometric p-value across every pairwise comparison of signatures from 3 pooled datasets. We only considered the comparisons of pooled datasets that do not overlap in terms of samples, e.g. the comparison of the signatures from 'Des Loi' and 'Des Paw' is excluded to avoid any bias.

In our analysis, we have restricted ourselves to the same platform for training our classifiers, and use the same supervised protocol on all datasets. This allowed us to check whether the first two explanations apply. We evaluated the relative overlap of every combination of two signatures that were extracted from a single dataset, see Figure 9A. For these 15 comparisons, the majority of genes are exclusively part of one signature (mean overlap 1.8%). This clearly indicates that, within the context of the datasets studied here, Explanations 1 and 2 are not the likely causes of the limited overlap between the signatures from these six datasets.

#### Explanation 3

Using our functional enrichment analysis we checked Explanation 3. The enrichment patterns of the signatures from single datasets are largely disparate (Figure 7). Two of the six signatures are not enriched for any functional category. There is only one gene set (GO:0007049: cell cycle), which is enriched in three signatures, namely those derived from Desmedt *et al. *[4], Miller *et al. *[18], and Loi *et al. *[24]. When considering the set of enriched gene sets that overlap between every pair of signatures (Figure 9D), we observed a mean overlap of less than 1%. Taken together, this indicates that there is very little support for the third explanation in these six datasets.

#### Explanation 4

Breast cancer is well known to be a complex disease which manifests itself in a range of subtypes. As a result, differences in the clinical composition of different datasets might explain the different signatures. However, when creating a histogram of signature overlap within the ER positive/negative subgroups we also found that the majority of genes are only part of one signature. Thus, heterogeneity with respect to ER status does not explain the limited degree of overlap among the signatures from these six datasets. Of course, it could still be that differences in the composition with respect to another (unknown) clinical parameter partly explain the lack of overlap.

#### Explanation 5

Signatures that are derived from pooled datasets have been derived using more samples, thereby easing small sample size related difficulties. Therefore, we inspected the relative overlap between signatures derived from one, two and three datasets. To avoid any bias, we only consider pairs of pooled datasets that do not overlap in terms of samples. The histogram and p-values shown in Figure 9A–C represent the mean over all these combinations. The overlap becomes progressively larger for signatures that are extracted from larger datasets (single dataset: 1.8%, two pooled datasets: 8.1%, three pooled datasets: 13.1%). At the same time this overlap becomes more significant (single dataset: -*log*10(*p*) = 2.1, two pooled datasets: -*log*10(*p*) = 13.6, three pooled datasets: -*log*10(*p*) = 23.2). The same trends were observed on the artificial data, and the ER positive subgroup (results not shown). Next, we repeated this analysis by looking for overlap in terms of enriched gene sets (Figure 9D–F). Similarly, signatures derived from the pooled datasets show a higher degree of enriched gene sets that overlap. The distinguishing property of these signatures is the fact that they were derived from a larger dataset, i.e. more samples. Thus, we conclude that Explanation 5 is the most relevant explanation for the low degree of overlap in genes from signatures derived from these six datasets.

### Consensus signature across six datasets

**Figure 10:**
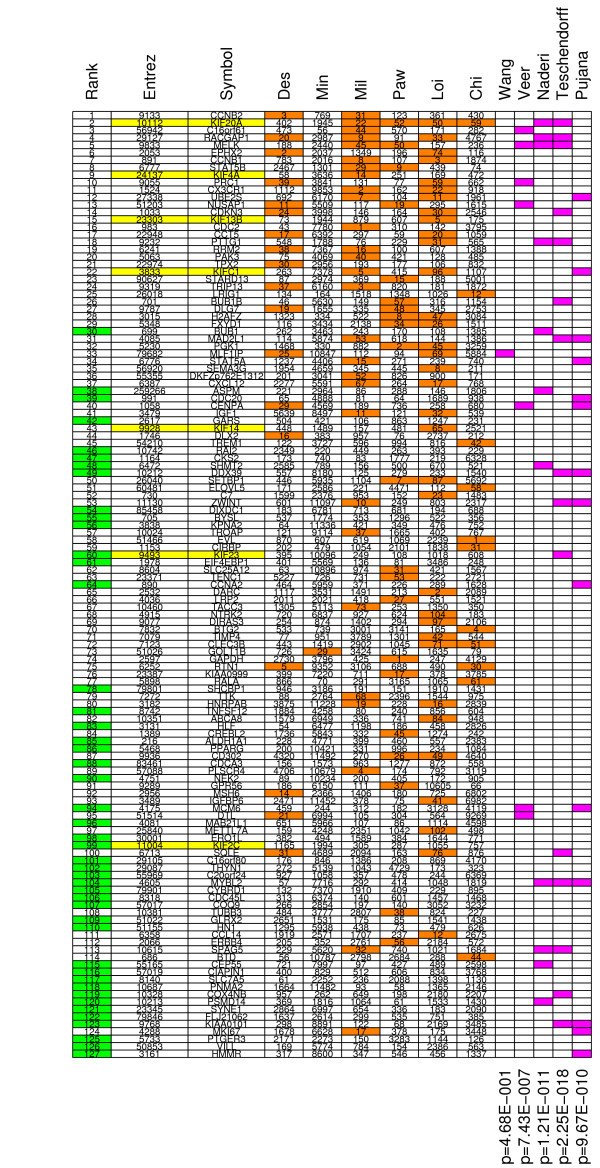
**Chart listing the 127 genes selected in the classifier trained on all six datasets.** For each gene, we list the rank, Entrez id, and Gene symbol. Green cell shading indicates the genes that are part of the signature from the six pooled datasets, which are not part of any of the signatures from the single datasets. Yellow cell shading indicates the seven microtubule associated genes. The succeeding columns indicate the rank position of a particular gene in each of the six separate rankings. An orange cell shading indicates the genes that were part of the individual signatures. The purple cell shading indicates the overlap to a group of existing breast cancer signatures (Wang *et al. *[2], Vijver *et al. *[3], Naderi *et al. *[19], Teschendorff *et al. *[20]), and a group of breast cancer associated genes (Pujana *et al. *[29]). P-values indicating the significance of the overlap (hypergeometric test) of these signatures is given at the bottom of the columns.

The signature from the six pooled datasets will be substantially different from those derived from each individual dataset. To gain insight into the difference, we created a chart indicating the rank position of all 127 genes selected in the classifier trained on all six datasets, along with rank positions from these genes in the rankings obtained from the individual datasets (Figure 10). Additional file 13 contains detailed information on the 127 genes, and the centroids of the classifier. Most of the 127 signature genes, had a much lower rank (larger rank number) in the individual signatures, implying that they're not part of those signatures. The difference in ranking of the 127 genes is especially large compared to the signature derived from the Minn *et al. *[22] and Chin *et al. *[25] data. This implies that those rankings are dominated by genes which are not generally relevant for breast cancer.

We compared our signature to signatures derived from different breast cancer datasets. We compared to the 70-gene signature from Wang *et al. *[2], 76-gene signature from Vijver *et al. *[3], 70-gene signature from Naderi *et al. *[19], and the 52-gene signature from Teschendorff *et al. *[20]. The latter of which was derived from the first three datasets. None of these three datasets were employed to derive the 127 gene signature. The overlap between the 127 genes and the Teschendorff signature is more significant (*p *= 2.3*e *- 18) than with Naderi (*p *= 1.2*e *- 11), Vijver (*p *= 7.4*e *- 7) or Wang (*p *= 4.7*e *- 1). This provides additional evidence that the limited signature overlap is most likely caused by small sample size difficulties.

The set of genes in the signature from all six datasets contains many well known and described genes. For instance, it confirms the significance of HMMR (Rank 127, Figure 10) which was only recently associated with breast cancer [29]. This gene was not picked up in any of the individual signatures. In the same study, Pujana *et al. *[29] derived a set of 164 genes that are associated with BRCA1, BRCA2, ATM, and CHEK2. This set of 164 genes is significantly overrepresented in the 127 signature genes (*p *= 2.4*e *- 9).

### Biological relevance of the consensus signature

Cell cycle and Mitosis pathways are the common background of most prognostic gene expression classifiers. Not surprisingly, many of the 127 genes have been previously described as markers of proliferation and poor prognosis in different types of cancer. These include: BUB1B, BUB1, MAD2L1, C16orf61 (DC13), CCT5, EIF4EBP1, MELK, MYBL2, CCNA2, CCNB1 and CCNB2. More specifically, EIF4EBP1 (4E-binding protein 1, 4E-BP1) is a EIF4E-binding protein that plays a critical role in the control of protein synthesis, survival, and cell growth. Rojo *et al. *[30] showed that 4EBP1 is activated in a high percentage of breast tumors, and is associated with higher malignant grade, tumor size, and local recurrence regardless of HER2/neu status (Armengol *et al. *[31]). Lin *et al. *[32] showed that MELK (maternal embryonic leucine zipper kinase), is overexpressed in both breast cancer specimens and cancer cell lines, and that its kinase activity possibly plays a significant role in breast cancer cell proliferation. They show that down-regulation of MELK by treatment with siRNA significantly suppressed the cell growth of breast cancer, indicating its crucial role in the proliferation of breast cancer.

C16orf61 (DC13) and CCT5 are examples of genes associated with therapy resistance. More specifically, C16orf61 (DC13) was previously found to be involved in multidrug resistance in breast cancer [33], and mRNA levels of CCT5 were significantly associated with resistance to docetaxel [34].

Cellular movement and cytokinesis pathways are significantly over-represented in our prognostic signature mainly through genes (KIF23, KIFC1, KIF14, KIF20A, KIF2C, KIF13B, KIF4A, PRC1, TUBB3, TPX2, CENP-A) linked to the microtubule motor activity (GO:0003777) and the microtubule-based movement (GO:0007018) GO categories. It is of major interest that these GO categories are non-significant for the range of pooling one to three datasets and are only picked up when pooling more datasets.

The genes that are associated with the microtubule categories are all part of the kinesin family. This family is composed of more than 40 genes involved in spindle assembly and function, chromosome segregation, mitotic checkpoint control and cytokinesis. They are motor proteins that generate movement along microtubules. KIF14 is a prognostic factor in breast and lung cancer (Corson *et al. *[35,36]). KIF20A is over-expressed in pancreatic ductal adenocarcinoma (PDAC) and siRNA knockdown of KIF20A expression in PDAC cell lines attenuated growth of those cells (Taniuchi *et al. *[37]). KIF2C is also known to be over-expressed in breast cancer cells. Treatment of breast cancer cells (T47D and HBC5) by siRNA against KIF2C suppressed KIF2C expression and inhibited the growth of the breast cancer cell lines (Shimo *et al. *[38]). Immunoprecipitation assay showed an interaction between PRC1 and KIF2C in breast cancer cells (Shimo *et al. *[38]). KIF4a is overexpressed in cervical cancer (Narayan *et al. *[39]). Treatment of lung cancer cells with siRNA for KIF4A suppressed the growth of these cells and induction of exogenous expression conferred cellular invasive activity on mammalians cells (Taniwaki *et al. *[40]). TUBB3 over-expression is a marker of poor prognostic in pancreatic, non-small cell lung carcinoma and ovarian cancer (Ferrandina *et al. *[41], Seve *et al. *[42,43], Lee *et al. *[44]) and is a potential marker of resistance to treatment by paclitaxel or vinorelbine-based chemotherapy. Kinesin related genes are currently vigorously pursued as therapeutic targets. Taxanes and Vinca alkaloids are major drugs in the clinical routine and are designed to bind and inhibit the motor function of the microtubules. The high frequency of side-effect of these drugs is due to the simultaneous alteration of microtubule cell-resting and differentiation function.

Drug companies are now putting an effort into the development of new molecules specifically targeting the kinesin family. Four other genes also present in the signature, TENC1, SEMA3G, CXCL12, and CX3CR1 also play a role in regulating cell motility and migration independently of the microtubule pathways (Chen *et al. *[45], Yu et al [46], Lopez-Bendito *et al. *[47], Lauro *et al. *[48]).

Taken together, this shows that many of the genes in the consensus signature have been characterized within a breast cancer con- text, and some have even been considered as drug targets. Nevertheless, not all of them have been covered, opening up possibilities for follow-up analysis. The fact that these genes have been derived from a compendium of six pooled datasets boosts confidence in their relevance within a clinical context.

## Conclusion

In our analysis we aimed to extract a robust classifier from multiple datasets. We explored the possibility of pooling datasets, which should alleviate small sample size related problems, and increase robustness. For a single dataset, we revisited the resampling strategy [5,13] which has been employed as a 'gold standard' for evaluating gene sets and has been applied to extract robust signatures, e.g. Yu *et al. *[14]. Our analysis provides proof that the resampling strategy does not alleviate potential problems due to sampling biases. Instead, by only taking the top *N *genes in every resampling into account, the obtained feature ranking is inferior to the ranking obtained based on all samples. Combining the statistics from each of the individual resamplings (either SNRs or Ranks), provides a ranking which is equivalent to the ranking based on all samples. Therefore, we propose to use the ranking over all samples in order to train a final classifier. 

Concurrent analysis of multiple datasets is an established way to increase statistical power, and thereby robustness. Pooling the datasets prior to any analysis would enable the largest gain in statistical power. We presented an example of pooling artificial datasets, which unveiled a significant negative correlation between the number of datasets which is pooled, and the DLCV error/validation error. We also observed a significant positive correlation between the degree of pooling and the number of genes which is selected. Thus, on the artificial data there is a clear synergetic effect associated with pooling of datasets.

Similarly, pooling breast cancer datasets represents a way to improve classification performance, since the pooled datasets will provide a better resemblance to the true underlying breast cancer population. For instance, breast cancer subtypes that are not present in one dataset might be present in another. A classifier trained on a pooled dataset was hypothesized to be more robust when applied to unseen, independent data. To test this hypothesis, we used a collection of six independent datasets, all measured on the same platform. Indeed, we observed a lower classification error for classifiers trained on pooled datasets. This significant trend is supported by a validation on a seventh independent dataset. The trends observed for pooling datasets strongly confirm the belief that there is a synergetic effect between pooling datasets and classification performance. Recently, another group [21] has come to a similar conclusion, albeit using only three datasets for training, and one for testing. A shortcoming in their analysis is that they do not switch the roles of which datasets are used for training and testing. Moreover, they mention the issue of small overlap among signatures, but do not provide any clear answers to that issue. Pooling in itself might lead to unwanted effects (i.e. Simpson's Paradox, [49]). Our results do not rule out the existence of detrimental effects due to pooling heterogeneous datasets, but imply that the synergetic effect of pooling is stronger than the detrimental effect of heterogeneity.

Signatures that have been extracted from independent cohorts of patients share only a limited number of genes. A variety of explanations have been proposed, only some of which have been thoroughly tested. We have checked whether there is any support for five of these explanations. On a collection of six breast cancer datasets, which were all measured on the same array, we applied the same supervised protocol. This lead to signatures that still lack overlap in terms of genes contained in the signatures. Similarly, our results point out that the overlap in terms of enriched pathways is just as limited, and reducing heterogeneity by stratifying based on ER status does not resolve the issue either. Of course, ER status might not be the only important source of heterogeneity. Therefore, we cannot completely rule out the effect of data heterogeneity on signature overlap. We believe that the limited signature overlap is most likely due to small sample size problems (Explanation 5). More specifically, that the variability between classifiers stems from the fact that the individual datasets are too small to capture the important sources of variation in the data. Moreover, pooling datasets might average-out adverse effects from heterogeneity that is only present in a subset of the samples/datasets. Explanation 5 is supported by a much larger overlap between signatures from pooled datasets than between signatures derived from single datasets. Nevertheless, we would like to point out that these conclusions might be specific to the six breast cancer datasets analyzed here and the specific techniques we employed to perform these analyses. That is, this conclusion may not extrapolate to different datasets/other tumor types.

Further evidence for the synergetic effect of pooling datasets comes from the functional enrichment analysis. We observed both low signal associations that become stronger, as well as categories that we can label as false positive associations since they're only found in one dataset and disappear when the complete set is analyzed. Moreover, we pick up relevant breast cancer associated genes in the signature derived from six pooled dataset, that are not picked up in any of the signatures from the separate datasets. Many of the genes in the signature have been previously characterized as drug targets and since our analysis is based on a large compendium it boosts our confidence in their potential relevance for clinical practice.

Overall, we hypothesize that the 127 gene signature that was derived from the six pooled datasets is one of the most robust signatures currently available. Our results indicate that new breast cancer datasets should not be analyzed in isolation, but should be analyzed in the context of the available compendium of breast cancer samples

## Methods

### Resampling procedure

The resampling strategy has been proposed for the derivation of robust signatures [5,13]. Briefly, the strategy works as follows. First, a subset of *s*% of the samples is selected, stratified relative to the outcome variable of interest. Second, for this subset all available genes *G *are ranked based on an appropriate selection statistic (e.g. the signal to noise ratio). Third, for each of *R *resamplings the top *N *genes are stored. The frequency of a gene being among the top *N *genes determines its final rank.

All genes with a non-zero frequency of appearing in the top *N *genes at least once can be considered part of the resulting robust signature [13]. However, when assessing a large number of resamplings, one would expect a fair number of genes to achieve a non-zero frequency by chance. To remove these genes, we propose to apply a threshold. Under the null hypothesis that there is no information in the dataset, we assume that each of the resamplings is a Bernouilli trial (with $p=\frac{N}{G}$). Under the null hypothesis, how often a gene appears in the top *N *across the *R *resamplings follows a Binomial distribution, with an expected value (*E*) of $E=\frac{RN}{G}$. Consequently, when assessing the frequency of occurrence in the top *N*, we divide by *R *again, leading to the following threshold *t*:

(1) $$t=\frac{N}{G}.$$

In our analysis, we used the resampling strategy in conjunction with the SNR (signal to noise ratio) as a ranking criterion. Assuming a dataset *A*, the absolute SNR is defined as:

(2) $$SNR(i)=\frac{\left|\mu (A_{i1})-\mu (A_{i2})\right|}{\sigma (A_{i1})+\sigma (A_{i2})}$$

where *SNR*(*i*) represents the SNR of gene *i*, *μ*(*A*_*i*1_) represents the mean of gene *i *for all samples in the class 1, and *σ*(*A*_*i*1_) represents the standard deviation of gene *i *for all samples in the class 1.

### Double loop cross validation

Wessels *et al. *[26] described a generally applicable framework for building diagnostic classifiers from high throughput data. We adopted this methodology combined with forward filtering as feature selector, the signal to noise ratio as criterion to evaluate the individual genes, and a nearest mean classifier (cosine correlation as distance measure). The training and validation procedure was performed employing 20 repeats of 5 folds cross validation in the outer (validation) loop, and 10 fold cross validation in the inner loop. Learning curves were constructed for up to 200 genes. At all points data splits were stratified with respect to the class prior probabilities.

The double loop cross validation method can be described in a few steps:

1. For each repeat, the data is split (stratified) into five parts (different splits for each repeat).

2. For each fold, four parts are used for the inner loop (training set), the fifth is used in the outer loop for validation (validation set).

3. On the training set data, a 10-fold cross validation is performed to estimate the optimal number of genes *n*.

4. Next, a classifier is trained on the complete training set, using the top *n *genes.

5. Finally, the performance of that classifier is assessed on the validation set.

6. After all repeats are completed, a final classifier is created by ranking the genes using all samples. The average number of *n *that were obtained are used to train the classifier. This classifier is then applied on an external validation set.

Typically, datasets are unbalanced with respect to the class priors. Moreover, the class priors will be different for different datasets. Hence, directly comparing overall error rates (fraction of wrong assignments), is not an appropriate comparative measure. Therefore, classification errors were calculated by using the average false positive false negative ratio (which takes the class priors into account), which is defined as:

(3) $$e_{FPFN}=\frac{\frac{FN}{TP+FN}+\frac{FP}{FP+TN}}{2}$$

where *e*_*FPFN *_represents the average false positive false negative ratio error, *TP *the number of true positives, and *TN *the number of true negatives. This ratio is equivalent to 1-.5(Sensitivity + Specificity). In each iteration in the inner loop, *n *is defined as the number of genes at which the *e*_*FPFN *_is minimal.

When we refer to the DLCV error, this is the validation error assessed in the outer loop of the protocol. In addition to the linear nearest mean classifier, we also performed the experiments using more complex, non-linear classifiers. To this end, we used a k-nearest neighbor classifier (K-NN) as well as a Support Vector Machine with a Radial Basis Function as kernel (SVM with RBF kernel). The K-NN classifier was run using *K *= 3 and the cosine correlation as distance measure. For the SVM we used the following parameters: *C *= 5 and *λ *= 2 (RBF kernel width). These two classifiers were used in conjunction with the same double loop cross validation protocol.

### Artificial dataset

An artificial dataset was used to be able to compare the ranking obtained by different strategies to a known ground truth. To this end, we generate a dataset which is comparable to microarray datasets in terms of dataset size. We constructed an artificial dataset with *M *samples and *G *genes. The data for each feature was sampled from the following two class conditional density functions:

(4) *P*(*X*|*ω*_1_) = *N*(*μ*(*j*), 1)

(5) *P*(*X*|*ω*_2_) = *N*(-*μ*(*j*), 1)

where

(6) $$\mu (j)=\{\begin{array}{lll} \mu_{0}-\frac{\mu_{0}(j-1)}{J} & \text{if} & 1\leq j\leq J,\\ 0 & \text{if} & J<j\leq G. \end{array}$$

The data that is generated using this model is more ideal than real-life microarray data, since it does not include any covariance terms. Artificial datasets were generated with the following setting of the parameters:

(7) *M *= 100 to 1000

(8) *G *= 5000

(9) *J *= {500, 2500, 5000}

(10) *μ*_0 _= .1

(11) *P*(*ω*_1_) = .5

(12) *P*(*ω*_2_) = .5

### Breast cancer datasets

The data from Veer *et al. *[1] was downloaded from the repository mentioned in the paper. Preprocessing was done as follows, the 24481 probes were pruned to 4919 by using only probes with at least a twofold change and *p *< 0.01 for three or more arrays [1].

We used a collection of six available datasets containing microarray data of breast cancer samples. These datasets were all measured on Human Genome HG U133A Affymetrix arrays and normalized using the same protocol. The datasets were downloaded from NCBI's Gene Expression Omnibus (GEO, ) with the following identifiers; GSE6532 [24], GSE3494 [18], GSE1456 [23], GSE7390 [4] and GSE5327 [22]. The Chin *et al. *[25] data set was downloaded from ArrayExpress (, identifier E-TABM-158).

To ensure comparability between the different datasets, they were all subjected to the same pre-processing procedure. Microarray quality-control assessment was carried out using the R AffyPLM package available from the Bioconductor web site (, [50]). We applied the Relative Log Expression (RLE) test and the Normalized Unscaled Standard Errors (NUSE) test. Chip pseudo-images were produced to assess artifacts on arrays that didn't pass the preceding quality control tests. Selected arrays were normalized according to a three step procedure using the RMA expression measure algorithm (, [51]): RMA background correction convolution, median centering of each gene across arrays separately for each data set and quantile normalization of all arrays.

For each sample, the ER status was determined by the expression profile [52]. This ensures a consistent assignment of the ER status across all samples, and rules out any inconsistencies in the assignments by pathologists. In addition, for part of the samples the IHC ER status was unavailable (see Additional file 14). All samples were ranked based on the expression level of the ER probe (probe 205225_at), after which we constructed an ROC curve using the IHC (Immuno-histo chemistry) assignment as true label. We then selected the threshold which resulted in the smallest error with regard to the true label (ERneg when 205225_at ≤ -1.84, ERpos when 205225_at > -1.84).

For the datasets from Desmedt, Miller, Pawitan, and Loi, there is an overlap in terms of samples. Part of these samples have been re-used, either with the same data (overlap Loi/Miller), or after a new hybridization (overlap Desmedt/Miller). We excluded all overlapping samples from the Desmedt and Loi datasets. This way, we ensured independence of the datasets, and avoid any bias.

We used the dataset from Vijver *et al. *[3] as an independent validation. This dataset was measured on the Agilent platform.

To validate the classifier trained on the six Affymetrix datasets on the Agilent dataset, we had to map the two probesets. We did this by mapping the probes from both platforms to the set of Entrez ids (11751) present on both. For the HG U133A array, only probes with an '_at', '_s_at', and '_x_at' extension were used (and prioritized in this order). When multiple probes match the same Entrez id (and had the same extension), the one with the highest variance was used.

Poor and good subgroups were defined as follows: Poor refers to samples with an event within five years, whereas Good refers to samples with event free survival and at least five years follow up.

Table 1 provides an overview of the datasets that were used.

### Functional enrichment analysis

For all signatures, we evaluated whether specific gene sets (i.e. functional groups), are overrepresented. We gathered a collection of 5480 gene sets from four databases: Gene Ontology (, Function and Process trees, 4745 gene sets), KEGG (,187genesets), Reactome (, 26 gene sets) and the MSDB (, C2, 522 gene sets). For the enrichment analysis we only used gene sets with at least five annotated Entrez IDs that are also present on the array, resulting in 1860 gene sets. We used the hypergeometric test to test the significance of the overlap between each signature and gene set. Multiple testing correction was taken into account by applying a Bonferroni correction (per signature).

## Authors' contributions

MHvV, LFAW, and MJTR contributed to the conceptual design of the study. MHvV and FR performed the analysis. MHvV, FR, HMH, and LFAW wrote the manuscript. HMH and MJvdV participated in the discussion of the results.

## Supplementary Material

Additional file 1 Information on summing SNRs. Click here for file

Additional file 2 Scatterplot indicating the classification error relative to the number of datasets that is pooled, using a K Nearest Neighbor classifier (K-NN, K = 3). A) DLCV error. B) Error on a large independent validation set of 2000 samples. The color corresponds to the number of datasets that was used. Labels indicate which combination of datasets was used. Click here for file

Additional file 3 Scatterplot indicating the classification error relative to the number of datasets that is pooled, using a Support Vector Machine classifier (SVM-RBF). A) DLCV error. B) Error on a large independent validation set of 2000 samples. The color corresponds to the number of datasets that was used. Labels indicate which combination of datasets was used. Click here for file

Additional file 4 Scatterplot indicating the classification error relative to the number of samples that is pooled. A) DLCV error. B) Error on the Vijver *et al. *[3] dataset. C) Number of genes selected by the DLCV protocol. The color corresponds to the number of datasets that was used. Labels indicate which combination of datasets was used. Click here for file

Additional file 5 Scatterplot indicating the classification error relative to the number of datasets that is pooled, using a K Nearest Neighbor classifier (K-NN, K = 3). A) DLCV error. B) Error on the Vijver *et al. *[3] dataset. The color corresponds to the number of datasets that was used. Labels indicate which combination of datasets was used. Click here for file

Additional file 6 Scatterplot indicating the classification error relative to the number of datasets that is pooled, using a Support Vector Machine Classifier (Radial Basis Function used as kernel). A) DLCV error. B) Error on the Vijver *et al. *[3] dataset. The color corresponds to the number of datasets that was used. Labels indicate which combination of datasets was used. Click here for file

Additional file 7 Network indicating the synergy between six real datasets (ER positive samples only). Each node represents a dataset, and each edge the effect on the DLCV error when pooling them. Four different effects were considered, synergy (bright green) when the pooled error is lower than each of the separate errors. Marginal synergy (light blue) when the pooled error is lower than the weighted mean of the separate errors, conversely marginal anti-synergy (yellow) when it is higher. Lastly, true anti-synergy (orange) indicates a higher DLCV error for the pooled dataset. Click here for file

Additional file 8 Scatterplot indicating the classification error relative to the number of datasets that is pooled (ER positive samples only). A) DLCV error. B) Error on the Vijver *et al. *[3] dataset. C) Number of genes selected by the DLCV protocol. The color corresponds to the number of datasets that was used. Labels indicate which combination of datasets was used. Click here for file

Additional file 9 Heatmap of the Bonferroni corrected p-values of the enrichment between each signature and a collection of gene sets (ER postive samples only). Only categories with at least 1 significant association are shown. Click here for file

Additional file 10 Network indicating the synergy between six real datasets (ER negative samples only). Each node represents a dataset, and each edge the effect on the DLCV error when pooling them. Four different effects were considered, synergy (bright green) when the pooled error is lower than each of the separate errors. Marginal synergy (light blue) when the pooled error is lower than the weighted mean of the separate errors, conversely marginal anti-synergy (yellow) when it is higher. Lastly, true anti-synergy (orange) indicates a higher DLCV error for the pooled dataset. Click here for file

Additional file 11 Scatterplot indicating the classification error relative to the number of datasets that is pooled (ER negative samples only). A) DLCV error. B) Error on the Vijver *et al. *[3] dataset. C) Number of genes selected by the DLCV protocol. The color corresponds to the number of datasets that was used. Labels indicate which combination of datasets was used. Click here for file

Additional file 12 Heatmap of the Bonferroni corrected p-values of the enrichment between each signature and a collection of gene sets (ER negative samples only). Only categories with at least 1 significant association are shown. Click here for file

Additional file 13 Centroids for the 127 gene classifier that was extracted from the six pooled datasets, including detailed info for the selected reporters. Click here for file

Additional file 14 Indication of the distribution of various clinical parameters. In all cases the number of samples (#), and percentage of samples (%) is indicated, except for the tumor size (represented as mm). Click here for file
