# Supplementary material for: Pooling breast cancer datasets has a synergetic effect on classification performance and improves signature stability
Source: BMC Genomics. 2008 Aug 6;9:375. doi: 10.1186/1471-2164-9-375 (PMC2527336; doi:10.1186/1471-2164-9-375)
Supplement: Additional file 1 — Information on summing SNRs. [file 1471-2164-9-375-S1.pdf]

Pooling breast cancer datasets has a synergetic effect on classification performance and improves signature stability Additional file 1

M.H. van Vliet*[1,2], F. Reyal[3,5], H.M. Horlings[3], M.J. van de Vijver[3,4] , M.J.T. Reinders[1] , L.F.A. Wessels[1,2]

[1]Information and Communication Theory Group, Faculty of Electrical Engineering, Mathematics and Computer Science, Delft University of Technology, Mekelweg 4, 2628 CD Delft, The Netherlands
[2]Bioinformatics and Statistics group, Department of Molecular Biology, Netherlands Cancer Institute, Plesmanlaan 121, 1066 CX Amsterdam, The Netherlands
[3]Department of Pathology, Netherlands Cancer Institute, Plesmanlaan 121, 1066 CX Amsterdam, The Netherlands
[4]Department of Pathology, Academic Medical Center, Meibergdreef 9, 1100 DD, Amsterdam, The Netherlands
[5]Department of Surgery, Institut Curie, 6 rue d'Ulm, 75005 Paris, France

Email: M.H. van Vliet*- M.H.vanVliet@TUDelft.nl;

*Corresponding author

Supplementary text S1: Summation of SNRs
Hypothesis: SNR of pooled dataset C is the sum of the SNRs of the separate datasets A and B (given some assumptions)

$$SNR_C = \frac{1}{2}SNR_A + \frac{1}{2}SNR_B \tag{1}$$

$$\frac{\mu(C_1) - \mu(C_2)}{\sigma(C_1) + \sigma(C_2)} = \frac{1}{2}\frac{\mu(A_1) - \mu(A_2)}{\sigma(A_1) + \sigma(A_2)} + \frac{1}{2}\frac{\mu(B_1) - \mu(B_2)}{\sigma(B_1) + \sigma(B_2)} \tag{2}$$

Assumptions

- The two datasets A and B have the same number of samples N; dataset C is the concatenation of A and B, and consequently has $2N$ samples,
- The two classes in each dataset have equal priors, the first subscript indicates the class (1 or 2), the second subscript indicates a sample.

For the numerator the following holds:

$$\begin{aligned}
\mu(C_1) - \mu(C_2) &= \frac{1}{2N}\sum_{i=1}^{2N} C_{1i} - \frac{1}{2N}\sum_{i=1}^{2N} C_{2i} && (3)\\
&= \frac{1}{2N}\sum_{i=1}^{N} A_{1i} + \frac{1}{2N}\sum_{i=1}^{N} B_{1i} - \frac{1}{2N}\sum_{i=1}^{N} A_{2i} - \frac{1}{2N}\sum_{i=1}^{N} B_{2i} && (4)\\
&= \frac{1}{2}\mu(A_1) + \frac{1}{2}\mu(B_2) - \frac{1}{2}\mu(A_2) - \frac{1}{2}\mu(B_2) && (5)
\end{aligned}$$

For the denominator the following holds:

$$\begin{aligned}
\sigma(C_1) + \sigma(C_2) &= \sqrt{E(C_1^2) - E(C_1)^2} + \sqrt{E(C_2^2) - E(C_2)^2} && (6)\\
& && (7)
\end{aligned}$$

First simplicity for 1 class only:

$$\begin{aligned}
\sigma(C_1)^2 &= E(C_1^2) - E(C_1)^2 && (8)\\
&= \frac{1}{2N}\sum_{i=1}^{2N}(C_{1i}^2) - (\frac{1}{2}\mu(A_1) + \frac{1}{2}\mu(B_1))^2 && (9)\\
&= \frac{1}{2N}(\sum_{i=1}^{N}(A_{1i}^2) + \sum_{i=1}^{N}(B_{1i}^2)) - (\frac{1}{2}\mu(A_1) + \frac{1}{2}\mu(B_1))^2 && (10)\\
&= \frac{1}{2N}(\sum_{i=1}^{N}(A_{1i}^2) + \sum_{i=1}^{N}(B_{1i}^2)) - \frac{1}{4}\mu(A_1)^2 - \frac{1}{4}\mu(B_1)^2 - \frac{1}{2}\mu(A_1)\mu(B_1) && (11)\\
&= \frac{1}{2}\left(\frac{1}{N}\sum_{i=1}^{N} A_{1i}^2 + \frac{1}{N}\sum_{i=1}^{N} B_{1i}^2 - \frac{1}{2}\mu(A_1)^2 - \frac{1}{2}\mu(B_1)^2 - \mu(A_1)\mu(B_1)\right) && (12)\\
&= \frac{1}{2}\left(\sigma(A_1)^2 + \sigma(B_1)^2 + \frac{1}{2}\mu(A_1)^2 + \frac{1}{2}\mu(B_1)^2 - \mu(A_1)\mu(B_1)\right) && (13)\\
&= \frac{1}{2}\left(\sigma(A_1)^2 + \sigma(B_1)^2 + \frac{1}{2}(\mu(A_1) - \mu(B_1))^2\right) && (14)
\end{aligned}$$

Substituting 5 and 14 in 2:

$$SNR_C = \tag{15}$$

$$\frac{\frac{1}{2}\mu(A_1) + \frac{1}{2}\mu(B_1) - \frac{1}{2}\mu(A_2) - \frac{1}{2}\mu(B_2)}{\sqrt{\frac{1}{2}\left(\sigma(A_1)^2 + \sigma(B_1)^2 + \frac{1}{2}(\mu(A_1) - \mu(B_1))^2\right)} + \sqrt{\frac{1}{2}\left(\sigma(A_2)^2 + \sigma(B_2)^2 + \frac{1}{2}(\mu(A_2) - \mu(B_2))^2\right)}} \tag{16}$$

$$\frac{\mu(A_1) + \mu(B_1) - \mu(A_2) - \mu(B_2)}{\sqrt{2\sigma(A_1)^2 + 2\sigma(B_1)^2 + (\mu(A_1) - \mu(B_1))^2} + \sqrt{2\sigma(A_2)^2 + 2\sigma(B_2)^2 + (\mu(A_2) - \mu(B_2))^2}} \tag{17}$$

If:

$$\mu(A_1) = \mu(B_1) \tag{18}$$

$$\mu(A_2) = \mu(B_2) \tag{19}$$

$$\sigma(A_1) = \sigma(B_1) \tag{20}$$

$$\sigma(A_2) = \sigma(B_2) \tag{21}$$

both 17 and the righthand side of 2 simplify to:

$$\frac{\mu(A_1) - \mu(A_2)}{\sigma(A_1) + \sigma(A_2)} \tag{22}$$
