# Supplementary material for: Pooling breast cancer datasets has a synergetic effect on classification performance and improves signature stability
Source: BMC Genomics. 2008 Aug 6;9:375. doi: 10.1186/1471-2164-9-375 (PMC2527336; doi:10.1186/1471-2164-9-375)
Supplement: Additional file 2 — Scatterplot indicating the classification error relative to the number of datasets that is pooled, using a K Nearest Neighbor classifier (K-NN, K = 3). A) DLCV error. B) Error on a large independent validation set of 2000 samples. The color corresponds to the number of datasets that was used. Labels indicate which combination of datasets was used. [file 1471-2164-9-375-S2.pdf]

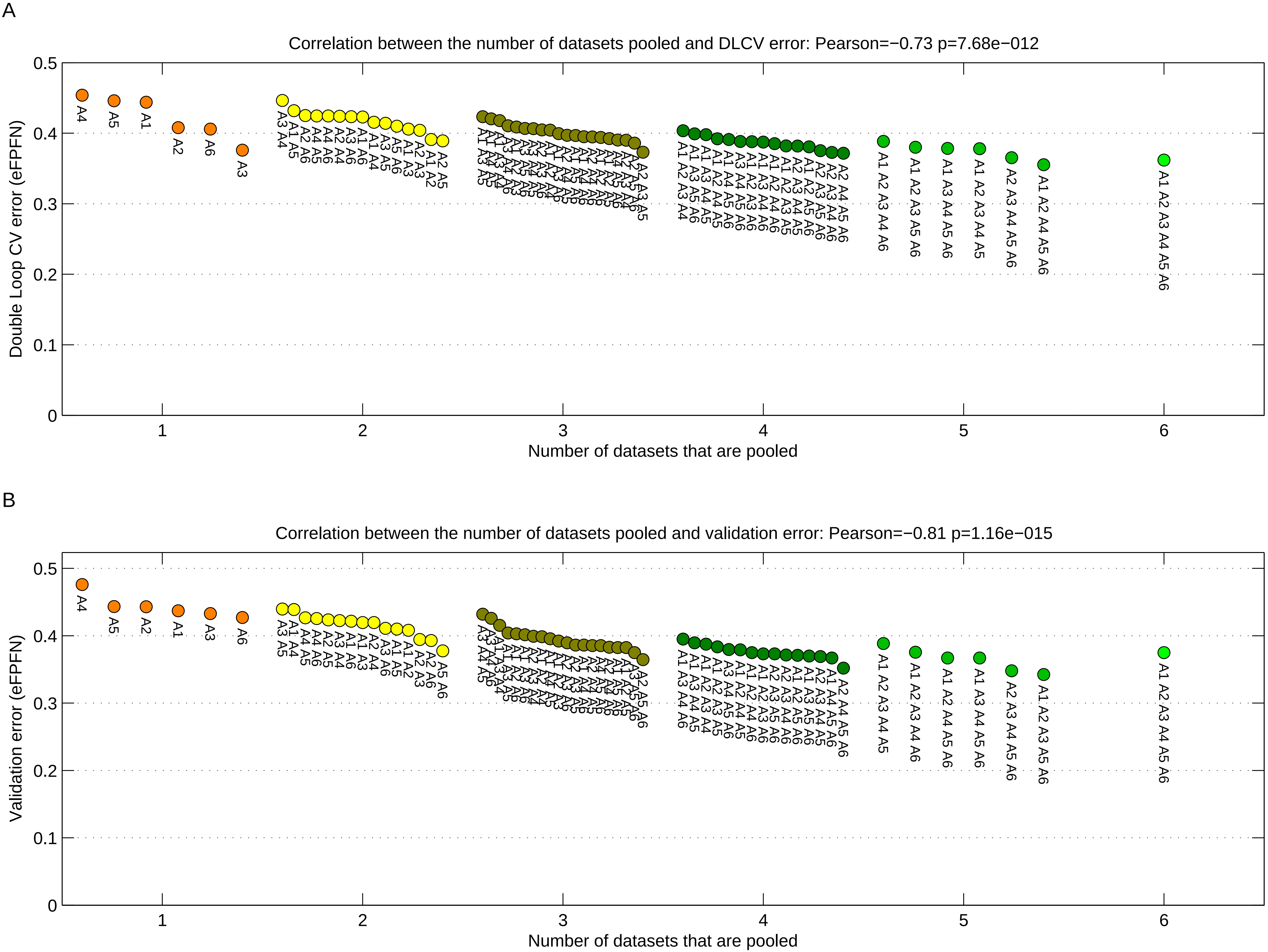
A
Correlation between the number of datasets pooled and DLCV error: Pearson=−0.73 p=7.68e−012
Double Loop CV error (eFPFN)
Number of datasets that are pooled
A4
A5
A1
A2
A6
A3
A1 A2 A3 A4 A6
A1 A2 A3 A5 A6
A1 A3 A4 A5 A6
A1 A2 A3 A4 A5
A2 A3 A4 A5 A6
A1 A2 A4 A5 A6
A1 A2 A3 A4 A5 A6
B
Correlation between the number of datasets pooled and validation error: Pearson=−0.81 p=1.16e−015
Validation error (eFPFN)
Number of datasets that are pooled
A4
A5
A2
A1
A3
A6
A1 A2 A3 A4 A5
A1 A2 A3 A4 A6
A1 A2 A4 A5 A6
A1 A3 A4 A5 A6
A2 A3 A4 A5 A6
A1 A2 A3 A5 A6
A1 A2 A3 A4 A5 A6
