# Supplementary material for: Pooling breast cancer datasets has a synergetic effect on classification performance and improves signature stability
Source: BMC Genomics. 2008 Aug 6;9:375. doi: 10.1186/1471-2164-9-375 (PMC2527336; doi:10.1186/1471-2164-9-375)
Supplement: Additional file 3 — Scatterplot indicating the classification error relative to the number of datasets that is pooled, using a Support Vector Machine classifier (SVM-RBF). A) DLCV error. B) Error on a large independent validation set of 2000 samples. The color corresponds to the number of datasets that was used. Labels indicate which combination of datasets was used. [file 1471-2164-9-375-S3.pdf]

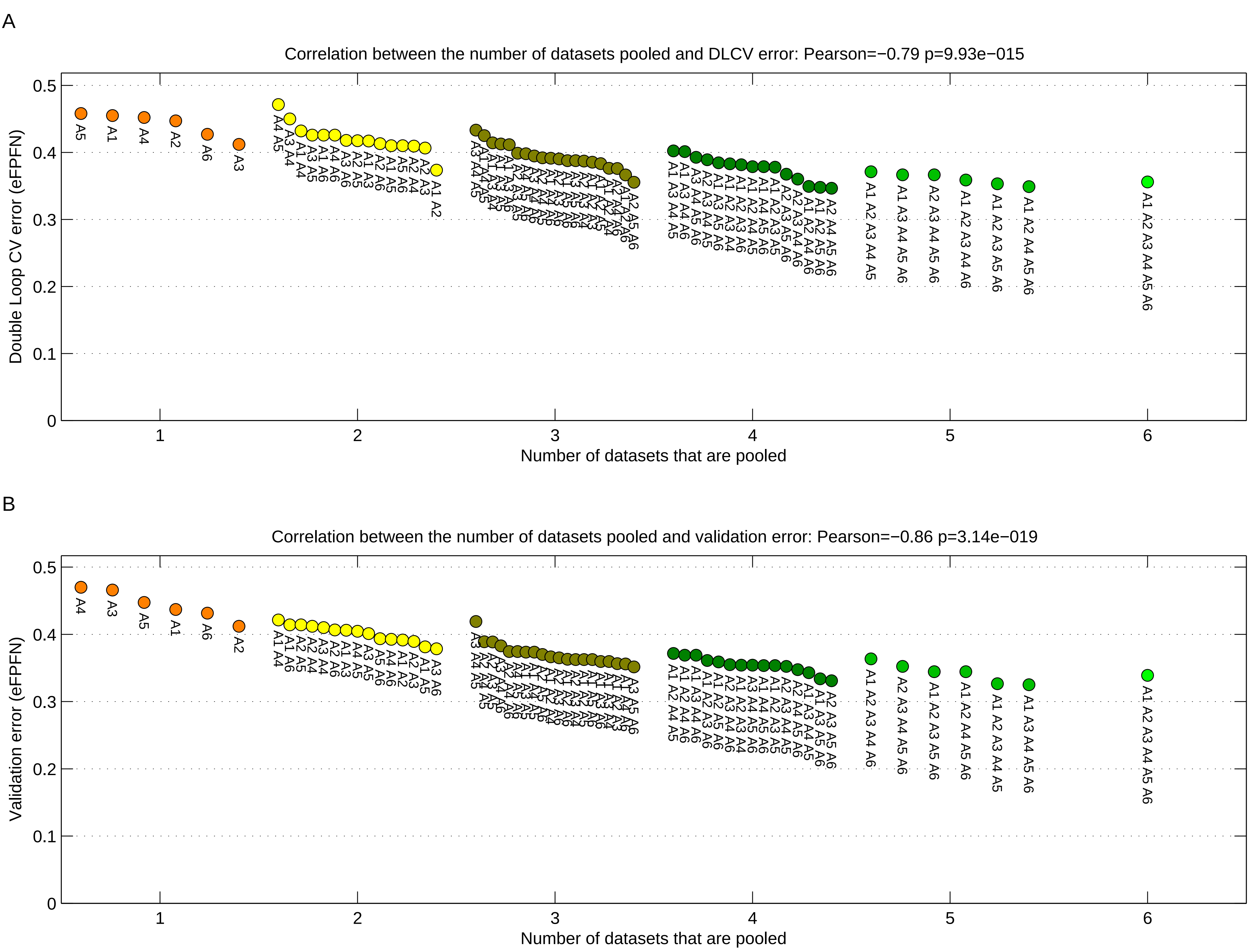

A
Correlation between the number of datasets pooled and DLCV error: Pearson=−0.79 p=9.93e−015
Double Loop CV error (eFPFN)
0.5
0.4
0.3
0.2
0.1
0
A5
A1
A4
A2
A6
A3
A4 A5
A3 A4
A1 A4
A3 A5
A1 A6
A4 A6
A3 A6
A2 A5
A1 A3
A2 A6
A1 A5
A5 A6
A2 A4
A2 A3
A1 A2
A3 A4 A5
A1 A4 A5
A1 A3 A4
A1 A3 A5
A1 A3 A6
A2 A3 A5
A4 A5 A6
A3 A4 A6
A2 A4 A5
A1 A4 A6
A2 A3 A6
A1 A5 A6
A3 A5 A6
A2 A3 A4
A1 A2 A3
A1 A2 A5
A1 A2 A4
A2 A4 A6
A1 A2 A6
A2 A5 A6
A1 A3 A4 A5
A1 A3 A4 A6
A3 A4 A5 A6
A2 A3 A4 A5
A1 A3 A5 A6
A1 A2 A3 A4
A1 A2 A3 A6
A1 A2 A4 A5
A1 A4 A5 A6
A1 A2 A3 A5
A2 A3 A5 A6
A2 A3 A4 A6
A1 A2 A4 A6
A1 A2 A5 A6
A2 A4 A5 A6
A1 A2 A3 A4 A5
A1 A3 A4 A5 A6
A2 A3 A4 A5 A6
A1 A2 A3 A4 A6
A1 A2 A3 A5 A6
A1 A2 A4 A5 A6
A1 A2 A3 A4 A5 A6
1
2
3
4
5
6
Number of datasets that are pooled
B
Correlation between the number of datasets pooled and validation error: Pearson=−0.86 p=3.14e−019
Validation error (eFPFN)
0.5
0.4
0.3
0.2
0.1
0
A4
A3
A5
A1
A6
A2
A1 A4
A1 A6
A2 A5
A2 A4
A3 A4
A2 A6
A1 A3
A4 A5
A3 A5
A5 A6
A4 A6
A1 A2
A2 A3
A1 A5
A3 A6
A3 A4 A5
A2 A4 A5
A2 A3 A5
A3 A4 A6
A2 A4 A6
A4 A5 A6
A1 A3 A5
A1 A4 A5
A2 A5 A6
A1 A2 A4
A1 A2 A6
A2 A3 A6
A2 A3 A4
A1 A2 A5
A1 A5 A6
A1 A3 A6
A1 A3 A4
A1 A2 A3
A1 A4 A6
A3 A5 A6
A1 A2 A4 A5
A1 A2 A4 A6
A1 A3 A4 A6
A1 A2 A3 A6
A1 A2 A5 A6
A2 A3 A4 A6
A1 A2 A3 A4
A3 A4 A5 A6
A1 A4 A5 A6
A1 A2 A3 A5
A2 A3 A4 A5
A2 A4 A5 A6
A1 A3 A4 A5
A1 A3 A5 A6
A2 A3 A5 A6
A1 A2 A3 A4 A6
A2 A3 A4 A5 A6
A1 A2 A3 A5 A6
A1 A2 A4 A5 A6
A1 A2 A3 A4 A5
A1 A3 A4 A5 A6
A1 A2 A3 A4 A5 A6
1
2
3
4
5
6
Number of datasets that are pooled
