# Supplementary figures and images for: Pooling breast cancer datasets has a synergetic effect on classification performance and improves signature stability
Source: BMC Genomics. 2008 Aug 6;9:375. doi: 10.1186/1471-2164-9-375 (PMC2527336; doi:10.1186/1471-2164-9-375)

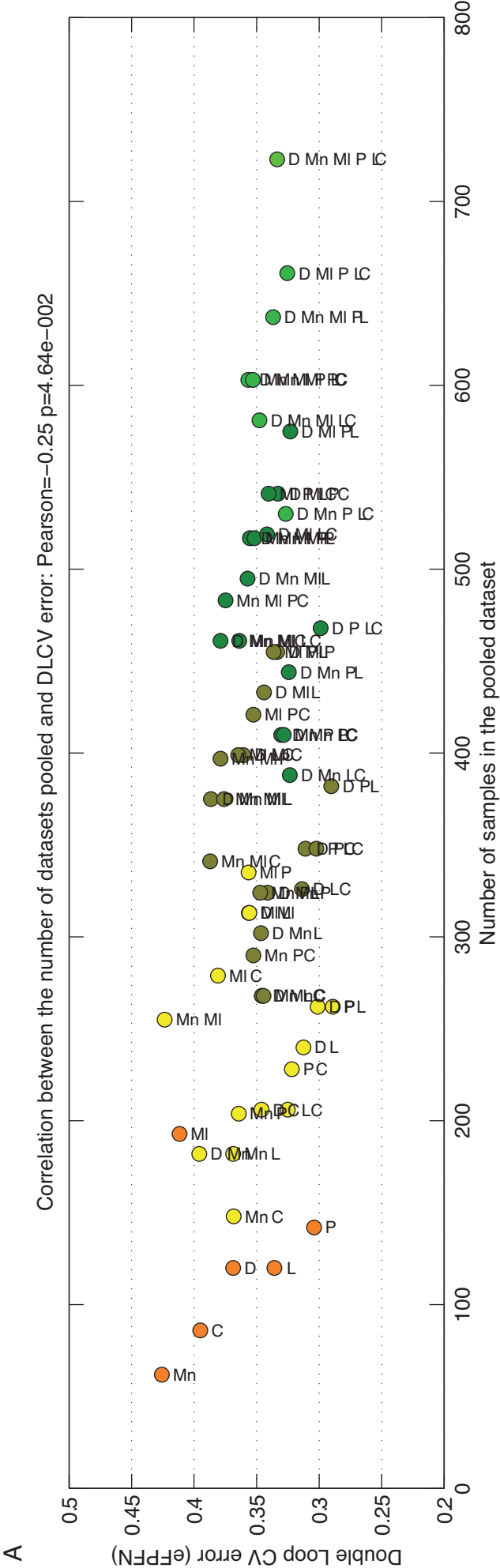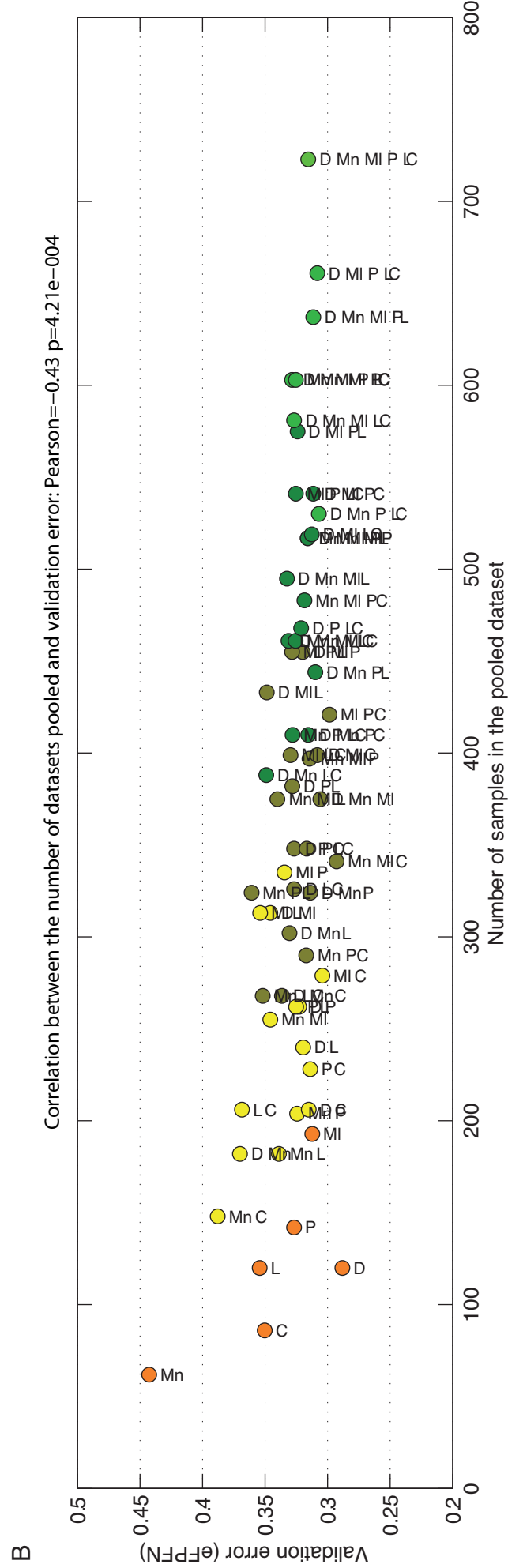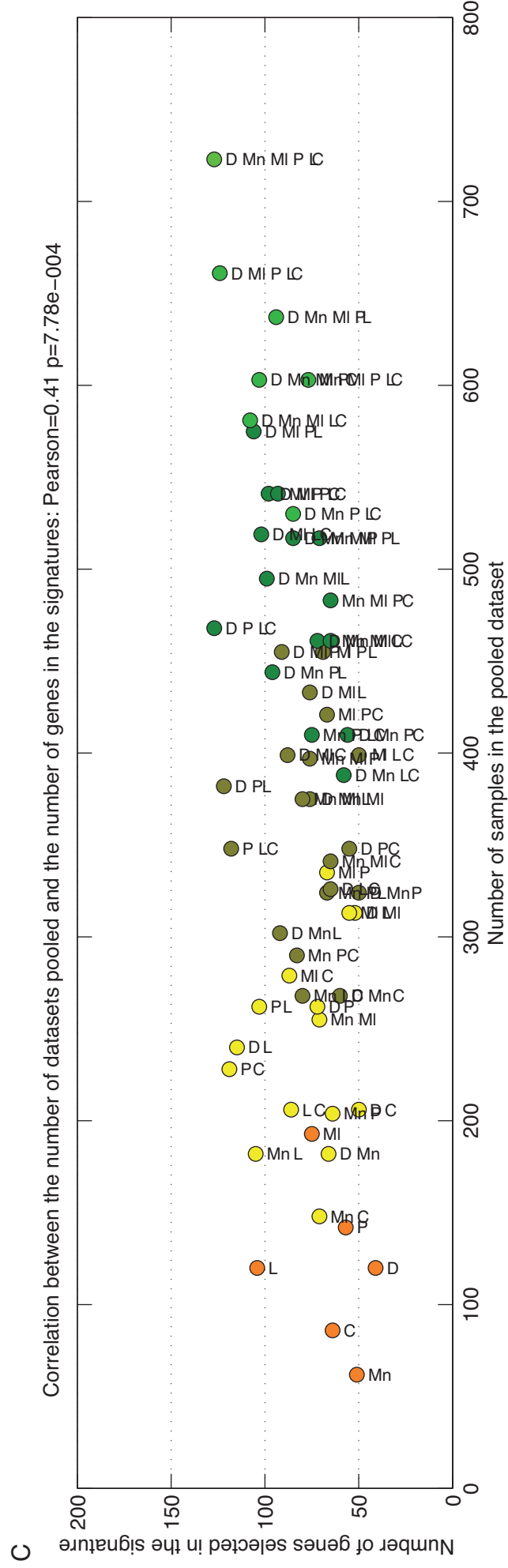

Supplement: Additional file 4 — Scatterplot indicating the classification error relative to the number of samples that is pooled. A) DLCV error. B) Error on the Vijver et al. [3] dataset. C) Number of genes selected by the DLCV protocol. The color corresponds to the number of datasets that was used. Labels indicate which combination of datasets was used. [file 1471-2164-9-375-S4.pdf]

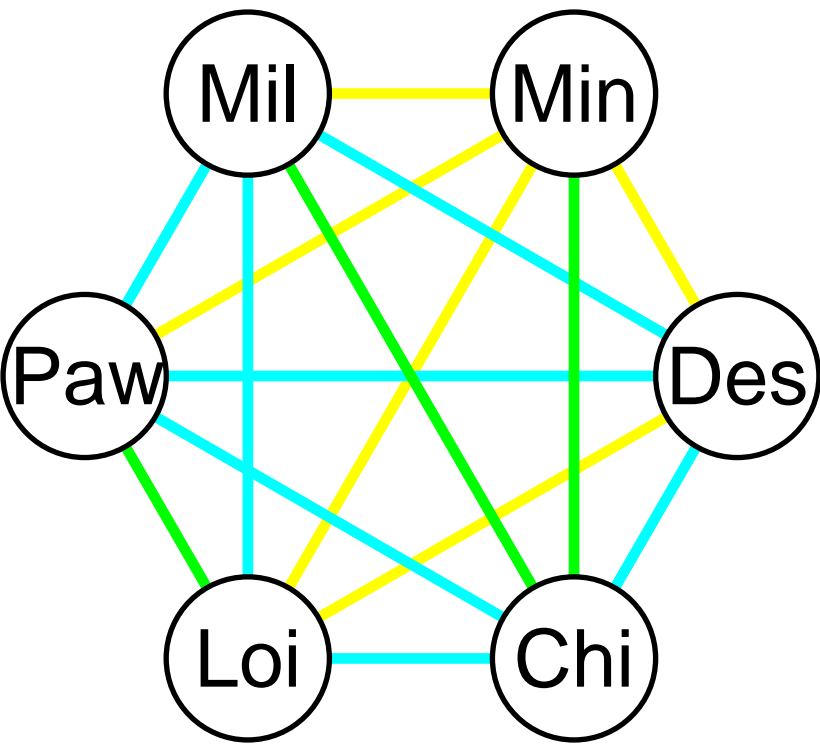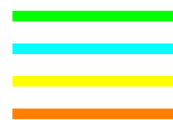

Synergy  
Marginal Synergy  
Marginal Anti-Synergy  
Anti-Synergy

Supplement: Additional file 7 — Network indicating the synergy between six real datasets (ER positive samples only). Each node represents a dataset, and each edge the effect on the DLCV error when pooling them. Four different effects were considered, synergy (bright green) when the pooled error is lower than each of the separate errors. Marginal synergy (light blue) when the pooled error is lower than the weighted mean of the separate errors, conversely marginal anti-synergy (yellow) when it is higher. Lastly, true anti-synergy (orange) indicates a higher DLCV error for the pooled dataset. [file 1471-2164-9-375-S7.pdf]

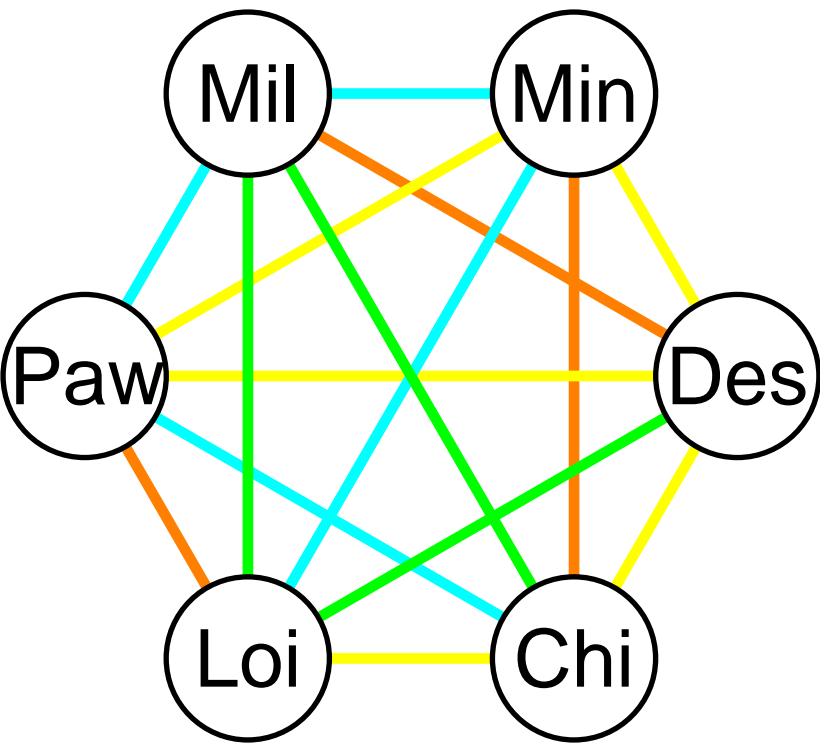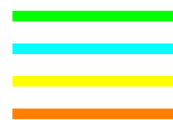

Synergy  
Marginal Synergy  
Marginal Anti-Synergy  
Anti-Synergy

Supplement: Additional file 10 — Network indicating the synergy between six real datasets (ER negative samples only). Each node represents a dataset, and each edge the effect on the DLCV error when pooling them. Four different effects were considered, synergy (bright green) when the pooled error is lower than each of the separate errors. Marginal synergy (light blue) when the pooled error is lower than the weighted mean of the separate errors, conversely marginal anti-synergy (yellow) when it is higher. Lastly, true anti-synergy (orange) indicates a higher DLCV error for the pooled dataset. [file 1471-2164-9-375-S10.pdf]
