# Supplementary material for: Pooling breast cancer datasets has a synergetic effect on classification performance and improves signature stability
Source: BMC Genomics. 2008 Aug 6;9:375. doi: 10.1186/1471-2164-9-375 (PMC2527336; doi:10.1186/1471-2164-9-375)
Supplement: Additional file 9 — Heatmap of the Bonferroni corrected p-values of the enrichment between each signature and a collection of gene sets (ER postive samples only). Only categories with at least 1 significant association are shown. [file 1471-2164-9-375-S9.pdf]

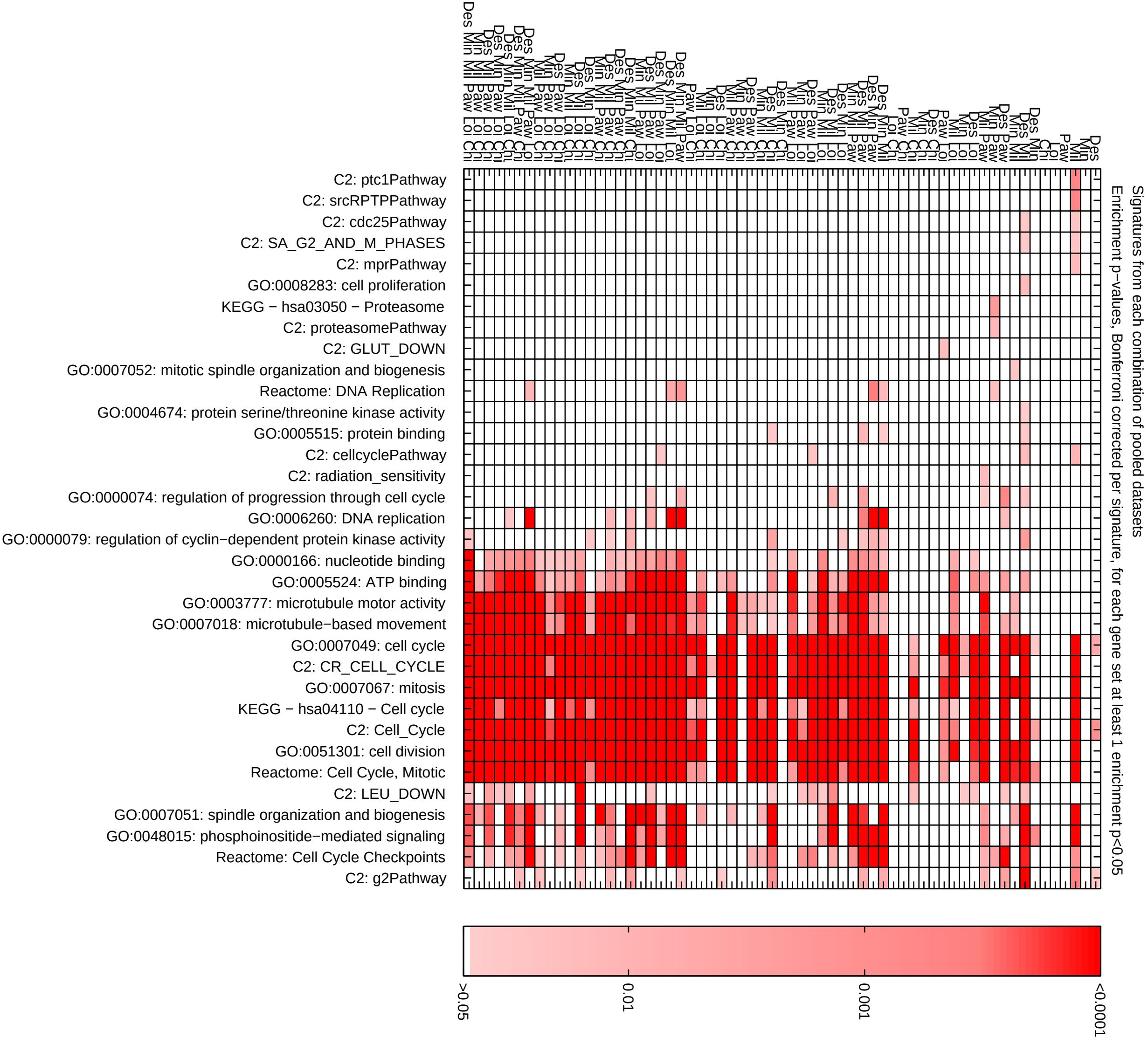

C2: ptc1Pathway
C2: srcRPTPPathway
C2: cdc25Pathway
C2: SA_G2_AND_M_PHASES
C2: mprPathway
GO:0008283: cell proliferation
KEGG − hsa03050 − Proteasome
C2: proteasomePathway
C2: GLUT_DOWN
GO:0007052: mitotic spindle organization and biogenesis
Reactome: DNA Replication
GO:0004674: protein serine/threonine kinase activity
GO:0005515: protein binding
C2: cellcyclePathway
C2: radiation_sensitivity
GO:0000074: regulation of progression through cell cycle
GO:0006260: DNA replication
GO:0000079: regulation of cyclin−dependent protein kinase activity
GO:0000166: nucleotide binding
GO:0005524: ATP binding
GO:0003777: microtubule motor activity
GO:0007018: microtubule−based movement
GO:0007049: cell cycle
C2: CR_CELL_CYCLE
GO:0007067: mitosis
KEGG − hsa04110 − Cell cycle
C2: Cell_Cycle
GO:0051301: cell division
Reactome: Cell Cycle, Mitotic
C2: LEU_DOWN
GO:0007051: spindle organization and biogenesis
GO:0048015: phosphoinositide−mediated signaling
Reactome: Cell Cycle Checkpoints
C2: g2Pathway
Signatures from each combination of pooled datasets
Enrichment p−values, Bonferroni corrected per signature, for each gene set at least 1 enrichment p<0.05
>0.05
0.01
0.001
<0.0001
