# Supplementary material for: Pooling breast cancer datasets has a synergetic effect on classification performance and improves signature stability
Source: BMC Genomics. 2008 Aug 6;9:375. doi: 10.1186/1471-2164-9-375 (PMC2527336; doi:10.1186/1471-2164-9-375)
Supplement: Additional file 12 — Heatmap of the Bonferroni corrected p-values of the enrichment between each signature and a collection of gene sets (ER negative samples only). Only categories with at least 1 significant association are shown. [file 1471-2164-9-375-S12.pdf]

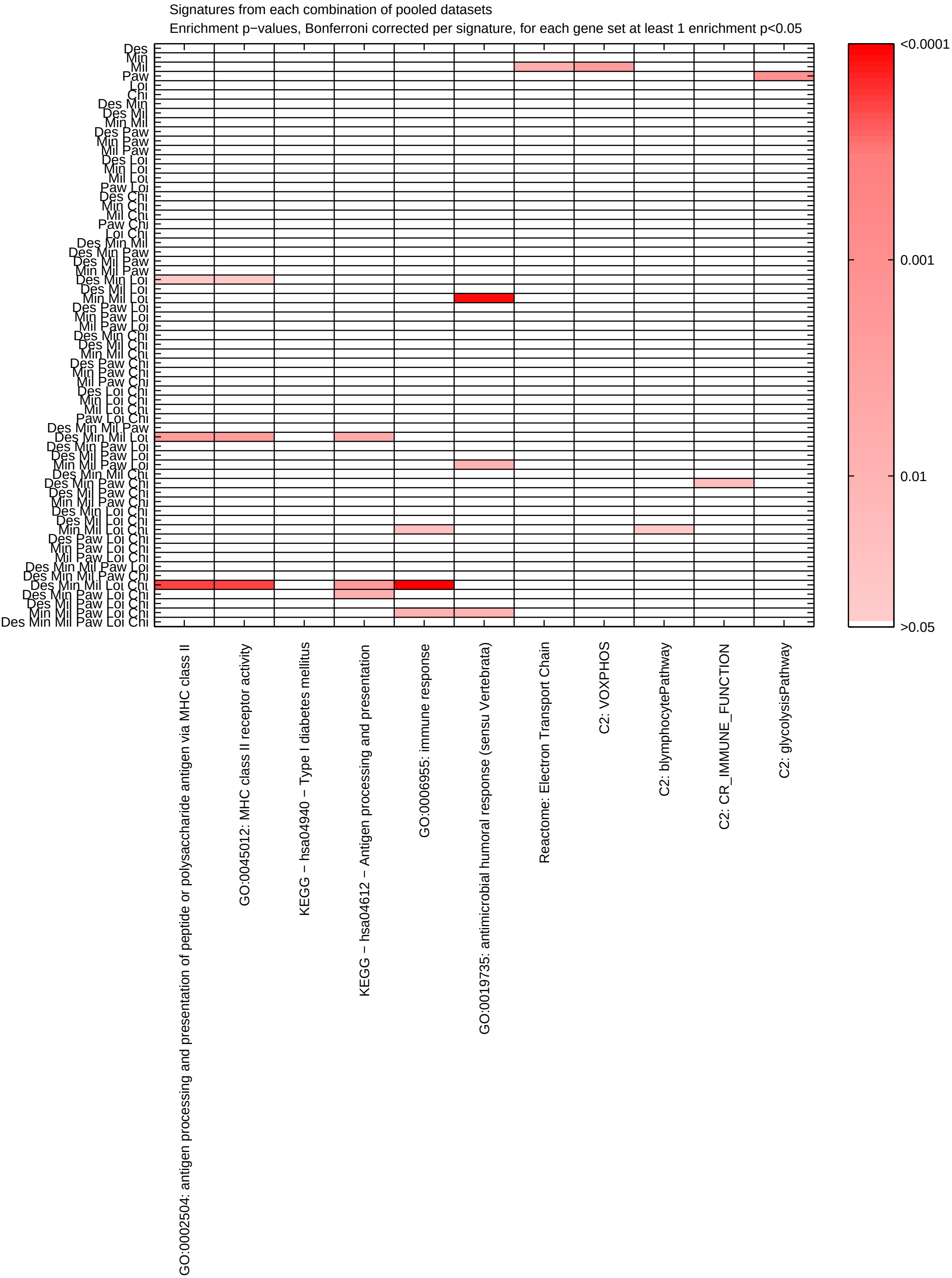
Signatures from each combination of pooled datasets
Enrichment p−values, Bonferroni corrected per signature, for each gene set at least 1 enrichment p<0.05
Des
Min
Mil
Paw
Loi
Chi
Des Min
Des Mil
Min Mil
Des Paw
Min Paw
Mil Paw
Des Loi
Min Loi
Mil Loi
Paw Loi
Des Chi
Min Chi
Mil Chi
Paw Chi
Loi Chi
Des Min Mil
Des Min Paw
Des Mil Paw
Min Mil Paw
Des Min Loi
Des Mil Loi
Min Mil Loi
Des Paw Loi
Min Paw Loi
Mil Paw Loi
Des Min Chi
Des Mil Chi
Min Mil Chi
Des Paw Chi
Min Paw Chi
Mil Paw Chi
Des Loi Chi
Min Loi Chi
Mil Loi Chi
Paw Loi Chi
Des Min Mil Paw
Des Min Mil Loi
Des Min Paw Loi
Des Mil Paw Loi
Min Mil Paw Loi
Des Min Mil Chi
Des Min Paw Chi
Des Mil Paw Chi
Min Mil Paw Chi
Des Min Loi Chi
Des Mil Loi Chi
Min Mil Loi Chi
Des Paw Loi Chi
Min Paw Loi Chi
Mil Paw Loi Chi
Des Min Mil Paw Loi
Des Min Mil Paw Chi
Des Min Mil Loi Chi
Des Min Paw Loi Chi
Des Mil Paw Loi Chi
Min Mil Paw Loi Chi
Des Min Mil Paw Loi Chi
GO:0002504: antigen processing and presentation of peptide or polysaccharide antigen via MHC class II
GO:0045012: MHC class II receptor activity
KEGG – hsa04940 – Type I diabetes mellitus
KEGG – hsa04612 – Antigen processing and presentation
GO:0006955: immune response
GO:0019735: antimicrobial humoral response (sensu Vertebrata)
Reactome: Electron Transport Chain
C2: VOXPHOS
C2: blymphocytePathway
C2: CR_IMMUNE_FUNCTION
C2: glycolysisPathway
<0.0001
0.001
0.01
>0.05
