# Supplementary material for: Pooling breast cancer datasets has a synergetic effect on classification performance and improves signature stability
Source: BMC Genomics. 2008 Aug 6;9:375. doi: 10.1186/1471-2164-9-375 (PMC2527336; doi:10.1186/1471-2164-9-375)
Supplement: Additional file 14 — Indication of the distribution of various clinical parameters. In all cases the number of samples (#), and percentage of samples (%) is indicated, except for the tumor size (represented as mm). [file 1471-2164-9-375-S14.pdf]

Pooling breast cancer datasets has a synergetic effect on classification performance and improves signature stability Additional file 14

M.H. van Vliet*[1,2], F. Reyal[3,5], H.M. Horlings[3], M.J. van de Vijver[3,4] , M.J.T. Reinders[1] , L.F.A. Wessels[1,2]

[1]Information and Communication Theory Group, Faculty of Electrical Engineering, Mathematics and Computer Science, Delft University of Technology, Mekelweg 4, 2628 CD Delft, The Netherlands
[2]Bioinformatics and Statistics group, Department of Molecular Biology, Netherlands Cancer Institute, Plesmanlaan 121, 1066 CX Amsterdam, The Netherlands
[3]Department of Pathology, Netherlands Cancer Institute, Plesmanlaan 121, 1066 CX Amsterdam, The Netherlands
[4]Department of Pathology, Academic Medical Center, Meibergdreef 9, 1100 DD, Amsterdam, The Netherlands
[5]Department of Surgery, Institut Curie, 6 rue d'Ulm, 75005 Paris, France

Email: M.H. van Vliet*- M.H.vanVliet@TUDelft.nl;

*Corresponding author

Dataset	Desmedt		Minn		Miller		Pawitan		Loi		Chin	
	#	%	#	%	#	%	#	%	#	%	#	%
Number of Samples	147		96		247		156		178		123	
Number of Poor	29	20	21	22	37	15	22	14	28	16	23	19
Number of Goor	91	62	41	43	156	63	120	77	92	52	63	51
ER Status (IHC) Positive	96	65	54	56	209	85	0	0	142	80	80	65
ER Status (IHC) Negative	51	35	42	44	34	14	0	0	30	17	43	35
ER Status (IHC) NA	0	0	0	0	4	2	156	100	6	3	0	0
ER Status (205225_at) Pos	101	69	63	66	184	74	124	79	139	78	87	71
ER Status (205225_at) Neg	46	31	33	34	63	26	32	21	39	22	36	29
HER2 Status (216836_s_at) Pos	20	14	13	14	33	13	18	12	18	10	17	14
HER2 Status (216836_s_at) Neg	127	86	83	86	214	87	138	88	160	90	106	86
Mean Tumor Size (mm)	23,3		36,4		22,5				23,9		26,5	
Tumor Size NA	0	0	0	0	0	0	156	100	4	2	2	2
Grade 1	24	16	0	0	65	26	28	18	27	15	14	11
Grade 2	58	39	0	0	126	51	57	37	74	42	43	35
Grade 3	65	44	0	0	54	22	60	38	36	20	62	50
Grade NA	0	0	96	100	2	1	11	7	41	23	4	3
Lymph Node Positive	0	0	62	65	82	33	0	0	37	21	68	55
Lymph Node Negative	147	100	34	35	156	63	0	0	135	76	55	45
Lymph Node NA	0	0	0	0	9	4	156	100	6	3	0	0
