# Supplementary material for: Pooling breast cancer datasets has a synergetic effect on classification performance and improves signature stability
Source: BMC Genomics. 2008 Aug 6;9:375. doi: 10.1186/1471-2164-9-375 (PMC2527336; doi:10.1186/1471-2164-9-375)
Supplement: Additional file 5 — Scatterplot indicating the classification error relative to the number of datasets that is pooled, using a K Nearest Neighbor classifier (K-NN, K = 3). A) DLCV error. B) Error on the Vijver et al. [3] dataset. The color corresponds to the number of datasets that was used. Labels indicate which combination of datasets was used. [file 1471-2164-9-375-S5.pdf]

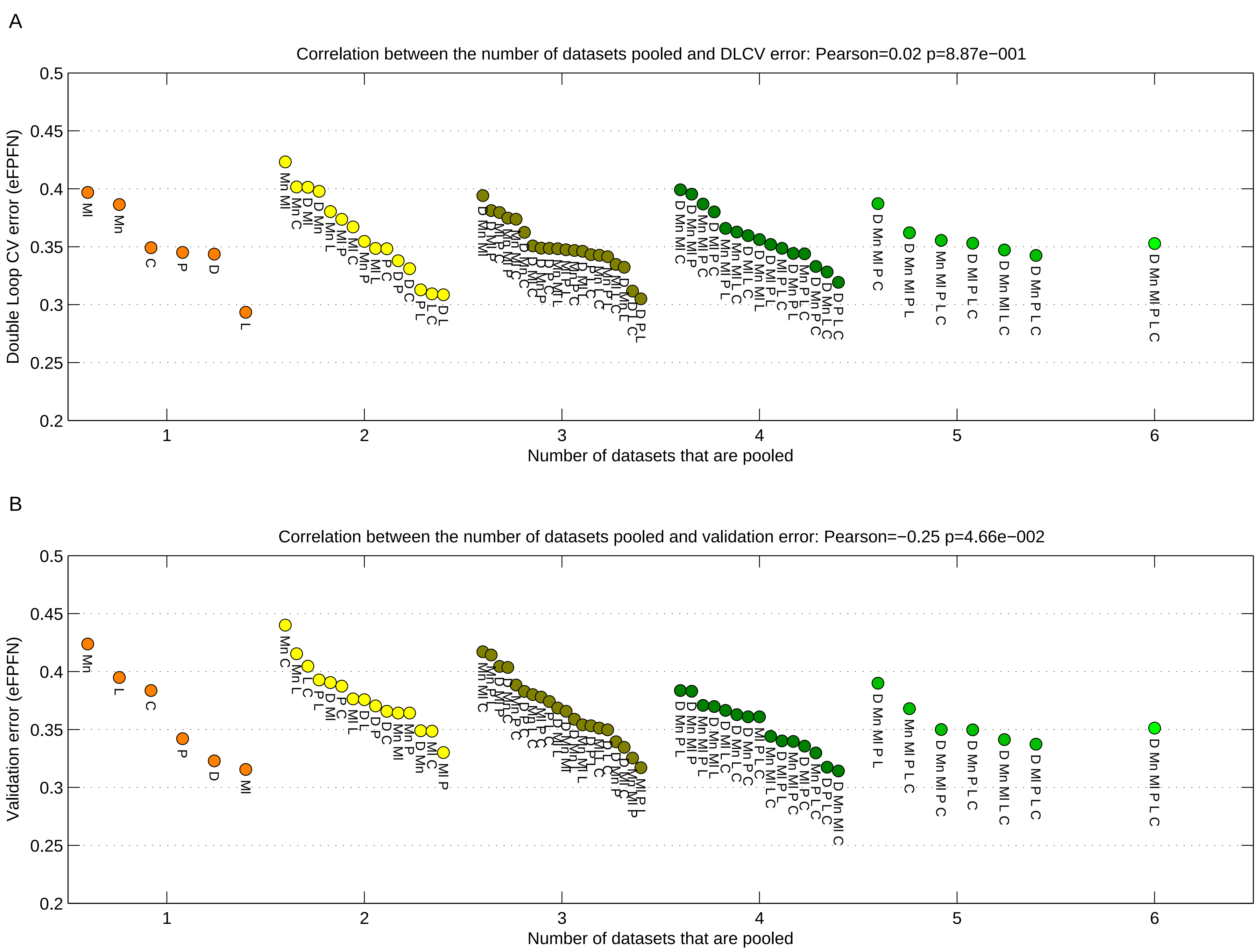
A
Correlation between the number of datasets pooled and DLCV error: Pearson=0.02 p=8.87e−001
Double Loop CV error (eFPFN)
Number of datasets that are pooled
B
Correlation between the number of datasets pooled and validation error: Pearson=−0.25 p=4.66e−002
Validation error (eFPFN)
Number of datasets that are pooled
