# Supplementary material for: Pooling breast cancer datasets has a synergetic effect on classification performance and improves signature stability
Source: BMC Genomics. 2008 Aug 6;9:375. doi: 10.1186/1471-2164-9-375 (PMC2527336; doi:10.1186/1471-2164-9-375)
Supplement: Additional file 6 — Scatterplot indicating the classification error relative to the number of datasets that is pooled, using a Support Vector Machine Classifier (Radial Basis Function used as kernel). A) DLCV error. B) Error on the Vijver et al. [3] dataset. The color corresponds to the number of datasets that was used. Labels indicate which combination of datasets was used. [file 1471-2164-9-375-S6.pdf]

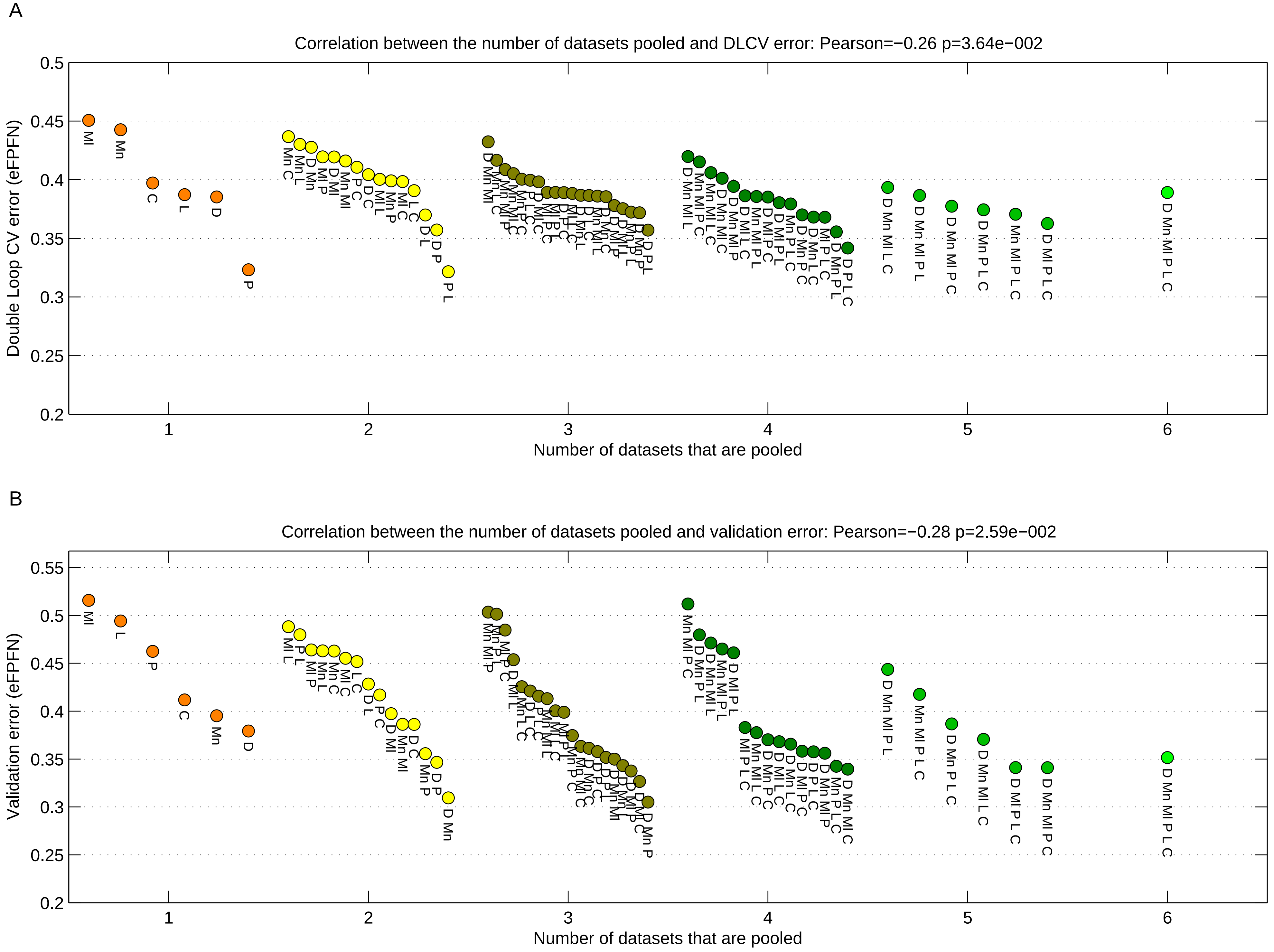
A
Correlation between the number of datasets pooled and DLCV error: Pearson=−0.26 p=3.64e−002
Double Loop CV error (eFPFN)
0.5
0.45
0.4
0.35
0.3
0.25
0.2
Ml
Mn
C
L
D
P
Mn C
Mn L
D Mn
Ml P
D Ml
Mn Ml
P C
D C
Ml L
Mn P
Ml C
L C
D L
D P
P L
D Mn Ml
Mn L C
Mn Ml P
Mn Ml C
Mn P C
P L C
D Ml C
Ml P C
Ml P L
D P C
Ml L C
D Mn L
D L C
Mn Ml L
D Mn C
D Ml P
D Ml L
Mn P L
D Mn P
D P L
D Mn Ml L
Mn Ml P C
Mn Ml L C
D Mn Ml C
D Mn Ml P
D Ml L C
Mn Ml P L
D Ml P C
D Ml P L
Mn P L C
D Mn P C
D Mn L C
Ml P L C
D Mn P L
D P L C
D Mn Ml L C
D Mn Ml P L
D Mn Ml P C
D Mn P L C
Mn Ml P L C
D Ml P L C
D Mn Ml P L C
1
2
3
4
5
6
Number of datasets that are pooled
B
Correlation between the number of datasets pooled and validation error: Pearson=−0.28 p=2.59e−002
Validation error (eFPFN)
0.55
0.5
0.45
0.4
0.35
0.3
0.25
0.2
Ml
L
P
C
Mn
D
Ml L
P L
Ml P
Mn L
Mn C
Ml C
L C
D L
P C
D Ml
Mn Ml
D C
Mn P
D P
D Mn
Mn Ml P
Mn P L
Ml P C
D Ml L
Mn L C
D L C
P L C
Mn Ml L
Ml L C
Ml P L
Mn P C
Mn Ml C
D Mn C
D P C
D P L
D Mn Ml
D Mn L
D Ml P
D Ml C
D Mn P
Mn Ml P C
D Mn P L
D Mn Ml L
Mn Ml P L
D Ml P L
Ml P L C
Mn Ml L C
D Mn P C
D Ml L C
D Mn L C
D Ml P C
D P L C
D Mn Ml P
Mn P L C
D Mn Ml C
D Mn Ml P L
Mn Ml P L C
D Mn P L C
D Mn Ml L C
D Ml P L C
D Mn Ml P C
D Mn Ml P L C
1
2
3
4
5
6
Number of datasets that are pooled
