# Supplementary material for: Pooling breast cancer datasets has a synergetic effect on classification performance and improves signature stability
Source: BMC Genomics. 2008 Aug 6;9:375. doi: 10.1186/1471-2164-9-375 (PMC2527336; doi:10.1186/1471-2164-9-375)
Supplement: Additional file 11 — Scatterplot indicating the classification error relative to the number of datasets that is pooled (ER negative samples only). A) DLCV error. B) Error on the Vijver et al. [3] dataset. C) Number of genes selected by the DLCV protocol. The color corresponds to the number of datasets that was used. Labels indicate which combination of datasets was used. [file 1471-2164-9-375-S11.pdf]

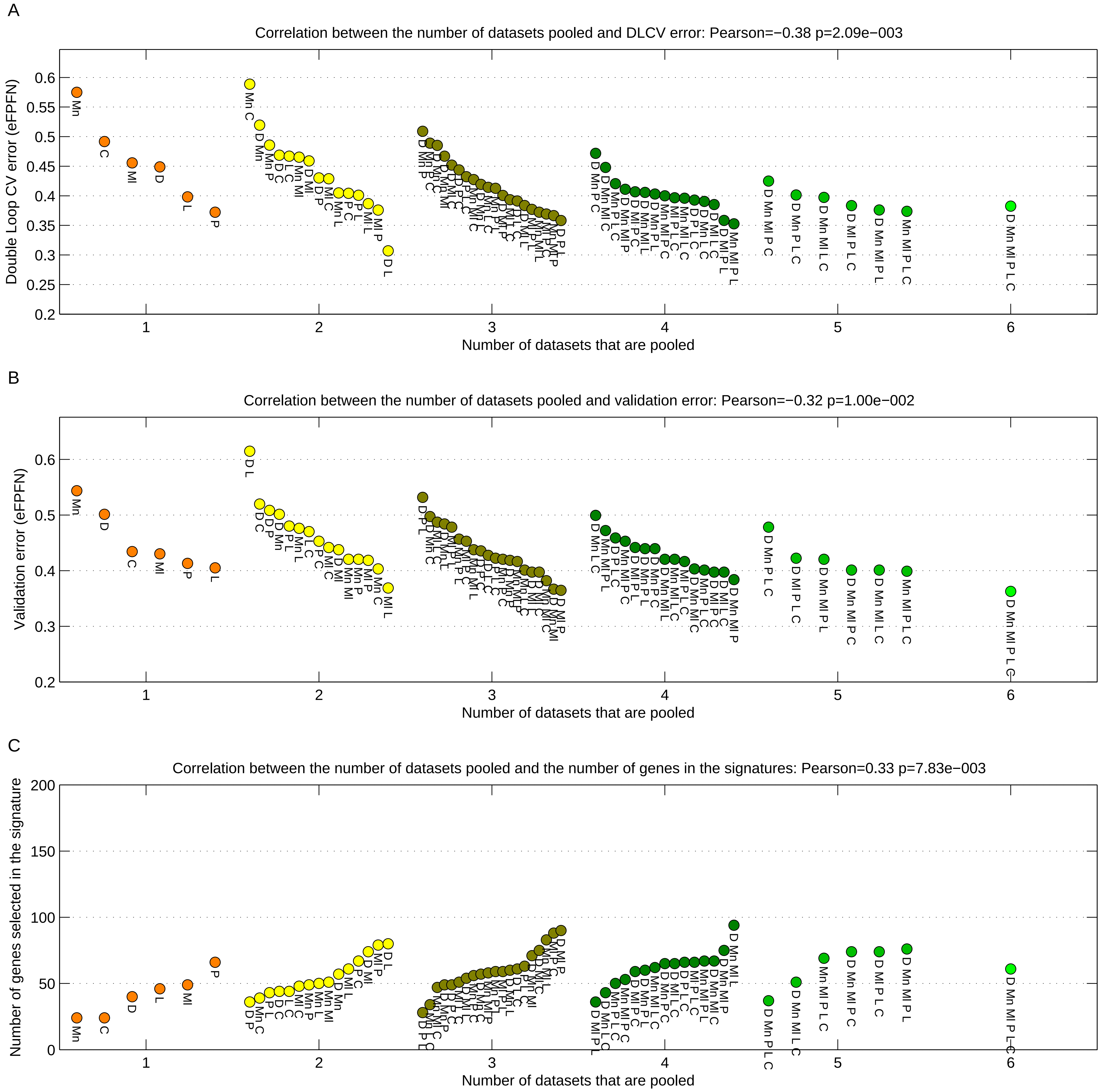
A
Correlation between the number of datasets pooled and DLCV error: Pearson=−0.38 p=2.09e−003
Double Loop CV error (eFPFN)
0.2 0.25 0.3 0.35 0.4 0.45 0.5 0.55 0.6
Number of datasets that are pooled
1 2 3 4 5 6
B
Correlation between the number of datasets pooled and validation error: Pearson=−0.32 p=1.00e−002
Validation error (eFPFN)
0.2 0.3 0.4 0.5 0.6
Number of datasets that are pooled
1 2 3 4 5 6
C
Correlation between the number of datasets pooled and the number of genes in the signatures: Pearson=0.33 p=7.83e−003
Number of genes selected in the signature
0 50 100 150 200
Number of datasets that are pooled
1 2 3 4 5 6
